# Exploring the Potential of Cytochrome P450 CYP109B1 Catalyzed Regio—and Stereoselective Steroid Hydroxylation

**DOI:** 10.3389/fchem.2021.649000

**Published:** 2021-02-18

**Authors:** Xiaodong Zhang, Yun Hu, Wei Peng, Chenghua Gao, Qiong Xing, Binju Wang, Aitao Li

**Affiliations:** ^1^ State Key Laboratory of Biocatalysis and Enzyme Engineering, Hubei Collaborative Innovation Center for Green Transformation of Bio-Resources, Hubei Key Laboratory of Industrial Biotechnology, School of Life Sciences, Hubei University, Wuhan, China; ^2^ State Key Laboratory of Physical Chemistry of Solid Surfaces, Collaborative Innovation Center of Chemistry for Energy Materials, National Engineering Laboratory for Green Chemical Productions of Alcohols, Ethers and Esters, College of Chemistry and Chemical Engineering, Xiamen University, Xiamen, China

**Keywords:** cytochrome P450, steroids hydroxylation, regioselectivity, stereoselectivity, redox partner, CYP109B1

## Abstract

Cytochrome P450 enzyme CYP109B1 is a versatile biocatalyst exhibiting hydroxylation activities toward various substrates. However, the regio- and stereoselective steroid hydroxylation by CYP109B1 is far less explored. In this study, the oxidizing activity of CYP109B1 is reconstituted by coupling redox pairs from different sources, or by fusing it to the reductase domain of two self-sufficient P450 enzymes P450RhF and P450BM3 to generate the fused enzyme. The recombinant *Escherichia coli* expressing necessary proteins are individually constructed and compared in steroid hydroxylation. The ferredoxin reductase (Fdr_0978) and ferredoxin (Fdx_1499) from *Synechococcus elongates* is found to be the best redox pair for CYP109B1, which gives above 99% conversion with 73% 15β selectivity for testosterone. By contrast, the rest ones and the fused enzymes show much less or negligible activity. With the aid of redox pair of Fdr_0978/Fdx_1499, CYP109B1 is used for hydroxylating different steroids. The results show that CYP109B1 displayed good to excellent activity and selectivity toward four testosterone derivatives, giving all 15β-hydroxylated steroids as main products except for 9 (10)-dehydronandrolone, for which the selectivity is shifted to 16β. While for substrates bearing bulky substitutions at C17 position, the activity is essentially lost. Finally, the origin of activity and selectivity for CYP109B1 catalyzed steroid hydroxylation is revealed by computational analysis, thus providing theoretical basis for directed evolution to further improve its catalytic properties.

## Introduction

Cytochrome P450 monooxygenases (CYPs) are heme-containing monooxygenase enzymes, which are broadly distributed among biological kingdoms and extensively involved in natural product biosynthesis, degradation of xenobiotics, steroid biosynthesis, drug metabolism etc. (Munro et al., 2007; Bernhardt and Urlacher, 2014; Li et al., 2020). They are considered to be the most versatile biocatalysts with the capability of catalyzing functionalization of non-activated hydrocarbons under mild reaction conditions in a regio- and stereoselective manner, thus accomplishing the challenging reactions that are difficult to be achieved in chemical approach (Nelson, 2018; Wang et al., 2020; Zhang et al., 2020b). Due to the above reasons, CYPs have attracted more and more attention with many CYPs discovered, identified, characterized, and investigated for many types of oxidations of a vast number of substrates, including hydroxylation (Guengerich, 2003; Lewis et al., 2011; Li et al., 2013; Li et al., 2015; Li et al., 2016a; Prier et al., 2017), alcohol oxidation, N-oxidation (Wang et al., 2014), N-, O-, S-dealkylation (Mallinson et al., 2018), and C-C bond cleavage (Urlacher and Girhard, 2019), as well as unusual reactions such as nitration of tryptophan, cyclopropanation (Coelho et al., 2013), and intramolecular C-H amination (Bernhardt et al., 2006; Isin and Guengerich, 2007).

Steroid-based drugs are the second largest marketed drugs after antibiotics, and CYPs catalyzed steroid hydroxylation is very important in pharmaceutical application due to the enhanced biological activity by this particular modification (Li et al., 2002; Bureik and Bernhardt, 2007; Donova, 2017). So far, a number of CYPs have been isolated from different origins (plants, animals and microorganisms) for steroid hydroxylation (Bureik and Bernhardt, 2007). Among them, microbial CYPs shows great superiority over the eukaryotic ones (Donova and Egorova, 2012; Li et al., 2020a). Because they can be overexpressed in high amounts in soluble form (Bernhardt and Urlacher, 2014) and are generally much more active with turnover numbers go from ten to a few hundred molecules per min. Therefore, more and more CYPs from different bacterial species capable of hydroxylating steroids with different regio- and stereoselectivity have been investigated, such as P450 families of CYP106, CYP154, CYP260, and CYP109 (Agematu et al., 2006; Arisawa and Agematu, 2007; Zhang et al., 2020a). Moreover, most of them have been employed and subjected to directed evolution to improve the both activity and selectivity for steroid hyxroxylation.

Nevertheless, for the enzyme P450 CYP109B1 from CYP109 family, although it has been reported many times for catalyzing broad spectrum substrates including fatty acids, primary *n*-alcohols and terpenoids, the use of it for steroid hydroxylation is far less explored (Furuya et al., 2008; Girhard et al., 2009; Girhard et al., 2010). And it was only used for testosterone hydroxylation to give 15β hydroxylated product in the presence of truncated adrenodoxin reductase (AdR) and adrenodoxin (Adx) from bovine adrenocortical mitochondria, but with very low activity (5%–10% conversion).

In this study, the potential of CYP109B1 catalyzed steroid hydroxylation was further explored. Frist, several pairs of electron transfer partners from different sources were employed and compared for achieving the best activity reproduction. Then, different substrates including four testosterone derivatives and four steroids bearing bulky substitutions at C17 position were catalyzed by CYP109B1 to explore its substrate scope. Finally, the origin of regio- and stereoselectivity of CYP109B1 catalyzed hydroxylation for different steroids was revealed, thus providing basis of further directed evolution on CYP109B1 to improve both activity and selectivity for practical industrial application.

## Materials and Methods

### Materials

Tryptone and yeast extract were purchased from OXOID (Shanghai, China), isopropyl β-D-1-thiogalactopyranoside (IPTG, >99%) and kanamycin sulfate (>99%) were bought from Sangon (Shanghai, China) and steroid compounds were purchased from Aladdin (Shanghai, China). All chemicals were of chemical purity and commercially available. Prime STAR Max DNA polymerase was bought from Takara (Shanghai, China), T5 super PCR Mix (Colony) DNA polymerase, DNA Maker, Trelidf™ Prestained Protein Ladder and TS-GelRed Nucleic acid dye were purchased from TSINGKE (Beijing, China). T5 exonuclease was obtained from New England Biolabs (Beverley, MA).

### Construction of Recombinant *E. coli* Cells as Whole-Cell Catalysts

Full length gene of CYP109B1 was amplified from the genome of *Bacillus subtilis* 168 (Kunst et al., 1997; Zhang et al., 2020) using specific upstream and downstream primers by polymerase chain reaction (PCR) (Supplementary Table S1), the resulted PCR fragment was ligated into the expression vector pRSFDuet-1 under control of T7 promoter using T5 exonuclease-dependent assembly approach (Xia et al., 2019). The plasmid pRSFDuet-1-CYP109B1 was transformed into *E. coli* BL21 (DE3) for protein expression and further characteristic assay.

In order to investigate the effect of different redox partners on the catalytic efficiency of CYP109B1, several pairs of redox partners from different sources were screened and optimized. First, the ferredoxin reductase Fdr_0978 and the ferredoxin Fdx_1499 from *Synechococcus elongates* PCC7942 (Plas et al., 1988; Sun et al., 2016) was linked with RBS *via* overlap PCR and integrally inserted into the second multiple cloning site (MCS2) of pRSFDuet-1-CYP109B1 followed by transformed into *E. coli* BL21 (DE3). In the same way, ferredoxin reductase FNR and ferredoxin Fd I from *spinach* (Aliverti et al., 1995; Binda et al., 1998; Mulo and Medina, 2017), and the ferredoxin reductase Fpr from *E. coli* (Bakkes et al., 2015; Bakkes et al., 2017) grouped with ferredoxin YkuN and ferredoxin YkuP from *B. subtilis* (Girhard et al., 2010), respectively, were also constructed for *in vivo* steroid hydroxylation. In addition, the plasmids pRSFDuet-1-CYP109B1-RhF and pRSFDuet-1-CYP109B1-BM3 carrying the genes of fused enzymes were constructed by fusing the heme domain of CYP109B1 to the N-terminal of reductase domain of P450RhF from *Rhodococcus* sp. Strain NCIMB 9784 and P450BM3 from *B. megatherium*, respectively.

To express CYP109B1 and redox partners, a 5 mL preculture of recombinant *E. coli* (DE3) cells was grown overnight in LB medium containing 50 mg/mL kanamycin sulfate at 37°C (220 rpm). The seed culture was used to inoculate a 200 mL Terrific Broth (TB) medium in a 500 mL baffled flask at 37°C and 220 rpm. When the optical density (OD_600_) reached 0.6, Isopropyl-thio-β-D-galactopyranoside (IPTG) was added to give a final concentration of 0.2 mM for protein induction at 25°C and 200 rpm for 14 h. Cells were harvested by centrifugation at 4,000 rpm for 10 min and thallus was washed twice with potassium phosphate buffer (pH 8.0), then resuspended in the same buffer controlling OD_600_ around 20 followed by quick-frozen in liquid nitrogen until further use.

### Steroid Hydroxylation With Recombinant Whole-Cell Catalysts

The reaction mixtures consist of 5 mL cell suspension in potassium phosphate buffer (100 mM, pH 8.0) containing 1 mM steroid substrates (**1**-**8** dissolved in dimethyl formamide), 5% (v/v) glucose, 1 U 6-glucose-phosphate-dehydrogenase and 5% (v/v) glycerol. The reactions were initiated by the addition of 1 mM NADP^+^ at 25°C, 200 rpm for 9 h. After reaction, 500 μL reaction mixture was taken and the products were extracted with 500 μL ethyl acetate, the organic phase was obtained by centrifugation at 12,000 rpm, which was then evaporated before resuspended in an equal volume acetonitrile for HPLC analysis.

### Expression and Purification of CYP109B1 and Corresponding Redox Partners

All proteins fused to a N-terminal poly histidine tag were overexpressed in *E. coli* BL21 (DE3) cell and purified using Ni-NTA column. For the purification of CYP109B1, *E. coli* BL21 (DE3) strain harboring pRSFDuet-1-CYP109B1, was inoculated as described above. And cells were harvested by centrifugation and resuspended in 50 mM potassium phosphate buffer (pH 8.0) containing 500 mM NaCl and 10 mM imidazole, then disrupted by sonication on ice. The crude cell extract was prepared by removal of cell debris by centrifugation at 9,000 rpm for 30 min and the supernatant was filtered through 0.45 μm pore size filters. The resulting cell-free extract was loaded onto an immobilized metal ion affinity chromatography column that had been equilibrated with 50 mM potassium phosphate buffer (pH 8.0) containing 500 mM NaCl. The column was washed with 10 column volumes of 50 mM potassium phosphate buffer (pH 8.0) containing 500 mM NaCl and 20 mM imidazole in order to remove non-specifically bound protein. Subsequently, the tagged protein was eluted with 50 mM potassium phosphate buffer (pH 8.0) containing 500 mM NaCl, 100 mM imidazole. The concentration of CYP109B1 was examined by the CO difference spectrum analysis using an extinction coefficient of 91 mM^−1^ cm^−1^ (Supplementary Table S2 **;** Supplementary Figure S2). Analogously, the expression and purification of redox partners were similar as described above. And the concentrations of redox partners were calculated by Bradford assay, using bovine serum albumin as a standard. Protein purity and molecular weight was assessed by SDS-PAGE. All purified proteins were stored in −80°C after quick-frozen with liquid nitrogen for further assays.

### Kinetic Analysis of CYP109B1 Catalyzed Steroid Hydroxylation

To determine the kinetic parameters of CYP109B1 catalyzed steroid hydroxylation, reactions were performed in 250 μL sodium phosphate buffer (pH 8.0,100 mM) containing 1 μM CYP109B1, 4 μM Fdr_0978, 20 μM Fdx_1499, 5% glycerol, 5% glucose, 1 U 6-glucase-phosphate-dehydrogenase and 2 mM MgCl_2_, at 30°C, 750 rpm for 2 min. The final substrate concentration was ranged from 0 mM to 1.5 mM. And the reaction was started by the addition of NADPH to a final concentration of 1 mM followed by shaking at 30°C, 750 rpm for 5 min, and quenched with an equivalent volume of acetonitrile. The substrate/production formation rate was determined by HPLC analysis. The values of *V*_max_, *K*_m_, and *k*_*cat*_ were determined by plotting the substrate consumption rate vs. the corresponding substrate concentration using a hyperbolic fit in GraphPad Prism 8.0 software (La Jolla, CA, United States). And the kinetic parameters are displayed in Table 1 and Supplementary Figure S3.

**TABLE 1:** Kinetic parameters of CYP109B1 catalyzed steroids hydroxylation^a^.

Substrate	*K*_*m*_ (μM)	*k*_*cat*_ (min^−1^)	*k*_*cat*_/*K*_*m*_ (M^−1^ min^−1^)	NADPH consumption rate (μmol/μmol*min)	Coupling efficiency (%)^b^
1	83.7 ± 20.4	4.7 ± 0.3	5.6 × 10^4^	74 ± 4.8	11 ± 5.3
2	136.3 ± 18.4	4.3 ± 0.1	3.1 × 10^4^	48 ± 11.8	10 ± 1.5
3	193.1 ± 38.2	3.5 ± 0.2	1.8 × 10^4^	147 ± 34.3	4 ± 0.9
4	163.1 ± 38.4	11.8 ± 0.7	7.2 × 10^4^	67 ± 2.9	5 ± 2.7

^a^ Reaction conditions: Kinetic parameters: 1 μM enzyme, steroids (0–1.5 mM), NADPH regeneration system (5 g/L glucose-6-phosphate, 5 g/L glycerol, 1 unit glucose-6-phosphate dehydrogenase and 1 mM NADPH). Reactions were allowed to proceed in 5 min with shaking at 750 rpm at 30°C. NADPH coupling efficiency: 1 μM mixture enzyme, 1 mM testosterone, 2 mM NADPH. Reaction mixtures were incubated at 750 rpm at 30°C for 6 min.

^b^ Coupling efficiency was calculated as the amount of the product produced divided by the amount of NADPH consumed.

For the determination of coupling efficiency of CYP109B1 for different steroid substrates, reaction mixture containing 1 μM CYP109B1, 4 μM Fdr_0978, and 20 μM Fdx_1499, 1 mM substrate and 2 mM NADPH in potassium phosphate buffer (100 mM, pH 8.0). And reaction was performed at 30°C, 750 rpm for 6 min. Consumption of NADPH was measured by absorbance variation at 340 nm with UV-1800 Spectrophotometer and product formation was analyzed *via* HPLC by addition of equivalent volume of acetonitrile. The coupling efficiency was calculated by the ratio of the product concentration with consumption concentration of NADPH (Table 1).

### HPLC Analysis

The conversion analysis of steroids and corresponding products was performed *via* reversed phase HPLC technique using a Shimadzu LC2030C system equipped with an Agilent ZORBAX SB-C18 column (4.6 × 250 mm, 5 μm; Agilent Technologies, Santa Clara, CA, United States). CYP109B1 reaction mixtures were extracted with ethyl acetate and organic phase evaporates naturally followed by added equal volume chromatographic grade acetonitrile and then subjected to HPLC analyze. The steroids and corresponding products were eluted using a gradient method (Supplementary Table S3 **),** starting with a mobile phase consisting of methanol, acetonitrile and water in a ratio of 15:15:70 with a flow rate of 1.5 mL/min, injection volume of 10 μL and a temperature at 40°C.

### Preparation and Identification of Hydroxylated Products by CYP109B1

For preparation and identification of hydroxylated products from CYP109B1 catalzyed steroid hydroxylation, large-scale cultivation 1 L Terrific Broth (TB) medium in a 2 L baffled flask was carried out as described above. The reaction mixture of l L was incubated at 25°C 200 rpm for 10 h with pH adjustment (pH 8.0) during the reaction. After reaction, equal volume ethyl acetate was then added for products extraction, followed by centrifugation to obtain the organic phase which was evaporated to give the crude products. The crude products were carefully loaded on a silica gel column (200–300 mesh particle size, 3.0 cm × 60 cm) with dichloromethane and methanol as elution solvent. Using gradient elution, the ratio of methanol to dichloromethane gradually changed from 1:100 to 1:50, the fractions containing the product were combined and dried in a rotary vacuum system to obtain the pure product that was used for NMR characterization for structural identification.

### Molecular Dynamics (MD) Simulation and Molecular Docking

The initial CYP109B1 structure was taken from protein data bank (PDB ID: 4RM4) (Zhang et al., 2015). The steroid substrates were docked into the active site of heme domain using Auto-Dock Vina in Discovery Studio based on a pose of CYP109B1 after a brief molecular dynamic (MD) simulation. The MD simulations of CYP109B1-progesteron and CYP109B1-canrenone complexes were performed with GPU version of Amber 18 package (Case et al., 2018). Missing hydrogen atoms were added by module leap of Amber 18 (Case et al., 2018). The force field for the resting-state species were parameterized using the “MCPB.py” modeling tool (Li et al., 2016b). The general AMBER force field (GAFF) (Wang et al., 2004) was used for steroid substrates, while the partial atomic charges and missing parameters were obtained from the RESP method (Bayly et al., 1993), using HF/6–31G* level of theory. 18 Na^+^ ions were added into the protein surface to neutralize the total charges of the systems. Finally, the resulting systems were solvated in a cubic box of TIP3P (Jorgensen et al., 1983) waters extending up to minimum cutoff of 15 Å from the protein boundary. The Amber ff14SB force field (Maier et al., 2015) was employed for the protein in all of the MD simulations. The initial structures were fully minimized using combined steepest descent and conjugate gradient method. The systems were then gently annealed from 10 to 300 K under canonical ensemble for 0.05 ns with a weak restraint of 15 kcal/mol/Å. 1 ns of density equilibration were performed under isothermal-isobaric ensemble at target temperature of 300 K and the target pressure of 1.0 atm using Langevin-thermostat (Izaguirre et al., 2001) and Berendsen barostat (Berendsen et al., 1984) with collision frequency of 0.002 ns and pressure-relaxation time of 0.001 ns Further equilibration of the systems was allowed for 4 ns to get well settled temperature and pressure. After proper minimizations and equilibrations, a productive MD run of 50 ns was performed for all the complex systems.

### NMR Characterization of the Products

All Nuclear Magnetic Resonance (NMR) spectra were recorded on Agilent 400 MHz spectrometer equipped with a 1H-19F/15N-31P probe at 25°C. Samples (20 mg/mL) for NMR experiment were typically prepared in dimethyl sulfoxide (DMSO) or Deuterium generation of chloroform (CDCl) and NMR data were processed and analyzed with MestReNova 14.2. Full ^1^H and ^13^C assignments, including the stereospecific assignment of prochiral ^1^H were obtained based on NMR spectra from standard 1D experiments as well as ^1^H DQF-COSY, ^1^H,^1^H-NOESY and ^1^H,^13^C-HSQC correlation experiments. The ^1^H and ^13^C chemical shifts were further confirmed with the values from previous studies (Reetz et al., 2008; de Flines et al., 2015; Qiao et al., 2017).

## Results

### Expression, Purification and Spectral Characterization of CYP109B1

The recombinant expression of P450 CYP109B1 was performed in *E. coli* (BL21) as a soluble protein. Under the induction of 0.2 mM Isopropyl-thio-β-D-galactopyranoside (IPTG) for 14 h at 25°C, the thallus of CYP109B1 displayed typical brick red color of cytochrome P450 monooxygenase (Supplementary Figure S1). The protein purification of CYP109B1 was conducted by immobilized metal (Nickel) affinity chromatography with 6 × His tag, and the purified protein of CYP109B1 with deeper brick red color was obtained. The theoretical protein size of CYP109B1 is approximately 43.5 kDa with 396 gene-encoded residues, which is in accordance with practical size (Supplementary Figure S1). Protein concentration of the purified protein was estimated to be 172 μΜ based on dithionite reduced and CO- difference spectral assay with extinction coefficient of ε_450–490_ = 91 mM^−1^ cm^−1^ (Omura and Sato, 1964), and a typical peak at 450 nm as one of the major spectral characteristics of cytochrome P450 monooxygenase was also observed (Supplementary Figure S2).

### Reconstitution of P450 CYP109B1 Activity for Steroid Hydroxylation

**FIGURE 1:**
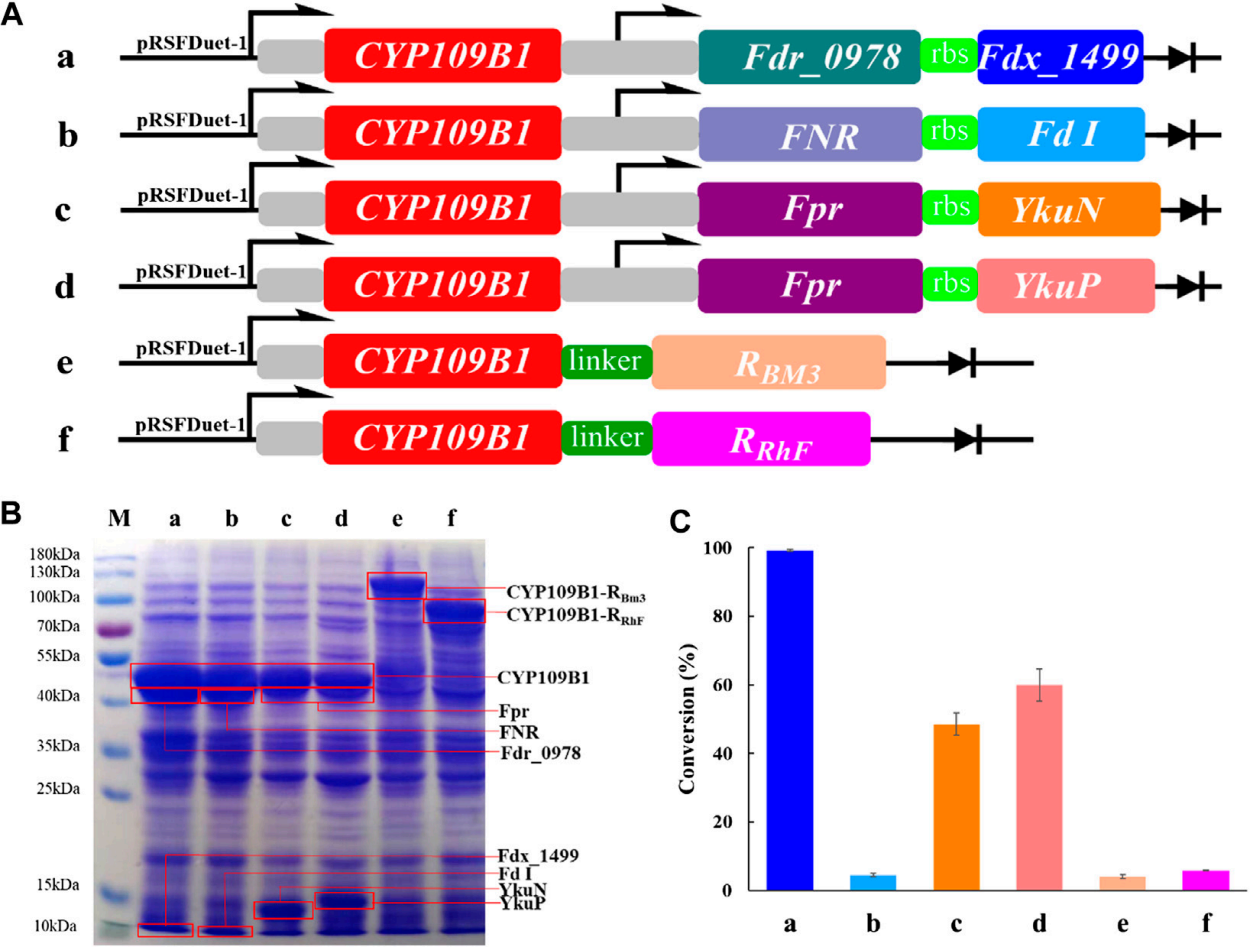
**(A):** The configuration of plasmids containing CYP109B1 with different pairs of redox partners (ferredoxin and ferredoxin reductase) or plasmids containing fused enzyme by fusing the heme domain of CYP109B1 to reductase domain of P450 BM3 or P450RhF. **a**: plasmid pRSFDuet-1 containing CYP109B1 and Fdr_0978/Fdx_1499 redox partner, **b**: plasmid pRSFDuet-1 containing CYP109B1 and FNR/Fd I redox partner from *spinach*, **c**: plasmid pRSFDuet-1 containing CYP109B1 and Fpr/YkuN, **d**: plasmid pRSFDuet-1 containing CYP109B1 and Fpr/YkuP, **e**: plasmid pRSFDuet-1 containing fused enzyme P450CYP109B1-BM3 in which heme domain of CYP109B1 was fused to the reductase domain of P450 BM3 from *Bacillus megaterium*, **f**: plasmid pRSFDuet-1 containing fused enzyme P450CYP109B1-RhFRed in which heme domain of CYP109B1 was fused to the reductase domain of P450RhF from *Rhodococcus* sp. Strain NCIMB 9784. **(B)**: SDS-PAGE analysis of recombination *E. coli* cells containing the corresponding plasmids in Figure 1A. **(C)**: Conversion of testosterone with whole-cell catalyst expressing different reconstituted catalytic system. Reaction conditions: recombinant *E. coli* cells frozen with liquid nitrogen was suspended in phosphate buffer (pH 8.0, 100 mM, OD_600_ =20) containing 1 unit glucose-6-phosphate dehydrogenase, 5 g/L glucose-6-phosphate, 5 g/L glycerol and 1 mM NADP^+^, reactions were conducted at 25°C, 200 rpm for 20 h.

In order to explore the potential of P450 CYP109B1 for steroid hydroxylation in drug synthesis, the electron transfer proteins flavodoxin reductase (Fpr) from *Escherichia coli,* flavodoxins (YkuN or YkuP) from *B. subtilis* (Girhard et al., 2010)*,* ferredoxin reductase (Fdr_0978) and ferredoxin (Fdx_1499) from *Synechococcus elongates* PCC7942 (Plas et al., 1988; Sun et al., 2016) as well as ferredoxin reductase (FNR) and ferredoxin (Fd I) from *spinach* (Aliverti et al., 1995; Binda et al., 1998; Mulo and Medina, 2017) were employed for the reconstitution of activity of CYP109B1. Based on different combination, four pairs of redox partners Fdr_0978/Fdx_1499, Fpr/YkuN, Fpr/YkuP and FNR/FdI were tested for their performance in CYP109B1catalyzed testosterone hydroxylation (Figure 1A **,** entries a-d). In addition, two chimeric proteins were also constructed by fusing the CYP109B1 to the reductase domain of P450RhF from *Rhodococcus* sp. Strain NCIMB 9784 or P450BM3 from *B. megatherium* (Figure 1A, entries f and g).

Next, the plasmids harboring the corresponding genes were individually transformed into recombinant *E. coli*, which resulted in six different whole-cell catalysts. The SDS-PAGE analysis was then performed to check the protein expression for either three-component catalytic system or self-sufficient catalytic system. As shown in Figure 1B, all the proteins were successfully expressed including the fused P450 enzymes. Subsequently, the whole-cell as catalysts were compared for steroid hydroxylation using testosterone as the model substrate. The results are presented in Figure 1C, the reconstituted catalytic system with CYP109B1 coupled to the redox pair of Fdr_0978/Fdx_1499 showed the best catalytic activity and 99% conversion was achieved to give 15β-hydroxylated product as main product with 78% selectivity (Supplementary Figure S4). In addition, when coupled with another two pairs of redox partners Fpr/ YkuN and Fpr/YkuP, obvious activity was also observed with substrate conversion being 49% and 60%, respectively (Supplementary Figure S4). One possible reason could be the lower protein expression of Fpr/YkuN and Fpr/YkuP compared with Fdr_0978/Fdx_1499 (Figure 1C). Although the natural redox partners of CYP109B1 have not been discovered, we have successfully identified a pair of redox partner ferredoxin reductase Fdr_0978 and ferredoxin Fdx_1499 from *Synechococcus elongates* PCC7942 which could efficiently deliver electrons from NADPH to CYP109B1 for steroid hydroxylation.

On the other hand, the two fused enzymes exhibited negligible activity, although proteins were well expressed. The result indicated that P450CYP109B1 is incompatible with the reductase domain of the self-sufficient P450RhFed and P450BM3 for the activity reproduction. In summary, with the support of heterogeneous redox partner ferredoxin reductase (Fdr_0978) and ferredoxin (Fdx_1499) from *Synechococcus elongates* PCC7942, P450 CYP109B1 showed excellent catalytic activity for steroid hydroxylation. Therefore, the reconstituted catalytic system of CYP109B1-Fdr_Fdx was selected for further study.

### P450 CYP109B1 Catalyzed Hydroxylation of Different Steroids

As a versatile P450 monooxygenase, P450CYP109B1 could accept various substrates including compactin, terpenoids like valencene, fatty acid and primary alcohols as good substrates (Girhard et al., 2009; Zhang et al., 2015). For the steroid hydroxylation, it was only reported that testosterone (Girhard et al., 2010) could be catalyzed by CYP109B1 to form the 15β-hydroxylated testosterone as main product in the presence of redox partners Adr/Adx from bovine adrenocortical mitochondria, but with very low activity (5–10% conversion). Afterward, although more steroids like androstenedione and norethindrone have also been identified to be catalyzed by CYP109B1 (Furuya et al., 2008), but the corresponding hydroxylated products have never been identified yet. Therefore, the constructed reconstituted catalytic system P450 CYP109B1-Fdr_Fdx with the highest catalytic efficiency were used in biotransformation of different steroid substrates.

**FIGURE 2:**
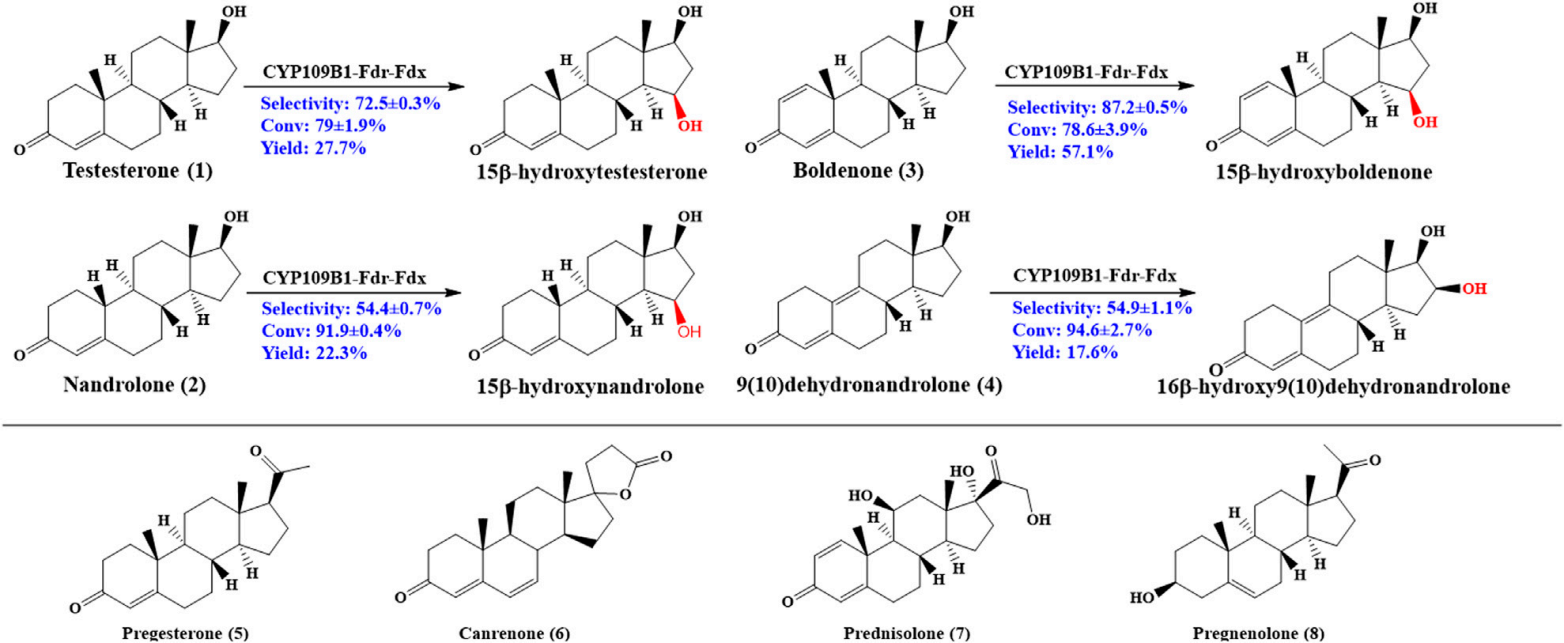
Regio- and stereoselective hydroxylation with CYP109B for eight different steroid substrates: testosterone (1), nandrolone (2), boldenone (3), 9 (10)-dehydronandrolone (4), progesterone (5), Canreone (6), Prednisolone (7) and Pregnenolone (8). Whole cell reaction of CYP109B1-Fdr_Fdx displayed catalyze activity for substrate 1 to 4**,** and major products were listed.

As shown in Figure 2, CYP109B1 showed excellent activity toward testosterone (1) and its derivatives nandrolone (2), boldenone (3) and 9 (10) dehydronandrolone (4). For substrates testosterone (1) and boldenone (3), good activity was also achieved with the 79% and 78% conversion, respectively. The main products produced was identified by HPLC/MS and NMR spectroscopy (data are listed in Supplementary Material) (Supplementary Figures S25-S41). In terms of selectivity, main hydroxylation occurs at 15β position for substrates 1 and 3, to give the 15β-hydroxylated product with above 70% selectivity (Supplementary Figures S5, S7) (Supplementary Figures S13, S16). And for substrate 2, the selectivity decreased to 54% due to the formation of 16β-hydroxylated nandrolone (29%) as minor product (Supplementary Figure S6) (Supplementary Figures S14, S15). By contrast, major hydroxylation happened at 16β position (55%) vs*.* 15β-hydroxylated product (29%) due to the different spatial structure of the substrate 4 (Supplementary Figure S8) (Supplementary Figures S17, S18). Obviously, C10 position of steroids **2** and **4** lack an angular methyl group comparted to 1 and 3, and molecular structural difference might lead to the decreased or switched selectivity. On the other hand, for those substrates with extra side substitution at C17 such as progesterone (5), canrenone (6), prednisolone (7) and pregnenolone (8), which are strictly rejected from active pocket of CYP109B1 and negligible activity was observed (Supplementary Figures S9-S12).

It was known that the corresponding product are interesting pharmaceutical intermediates for the production of anti-inflammatory, diuretic, anabolic, contraceptive, antiandrogenic, progestational, and antitumor drugs, or they are themselves biologically active (Fernandes et al., 2003; Furuya et al., 2009; Donova and Egorova, 2012). To the best of our knowledge, some of the steroidal C15 or C16 alcohols have not been reported to date, for example, 15β-hydroxynandrolone, 15β-hydroxyboldenone and 16β-hydroxy9 (10)dehydronandrolone. Thus, CYP109B1 has great potential as steroid hydroxylase and used in the production of high value-added steroids drugs in industrial application.

## Kinetic Study of CYP109B1 Catalyzed Steroid Hydroxylation

In order to make a further insight into the catalytic efficiency of CYP109B1 for steroid hydroxylation, kinetics and NADPH coupling efficiency for substrates 1-4 were determined. Measurements were carried out using pure P450CYP109B1 together with purified redox partners Fdr_0978 and Fdx_1499, a molar ratio of CYP109B1: Fdr: Fdx = 1: 4: 20 was employed and the results are listed in Table 1. Among the four substrates tested, P450 CYP109B1 exhibited the best affinity toward testosterone **1** and *K*_*m*_ value was estimated to be approximately 84 μΜ. While for catalytic performance, the best catalytic performance (*k*_*cat*_/*K*_*m*_) was obtained for substrate 4, which was measured to be 7.2 × 10^4^ M^−1^ min^−1^, indicating that 9 (10)-dehydronandrolone was the most preferable steroid substrate for CYP109B1 in terms of catalytic activity. In addition, coupling efficiency of CYP109B1 for substrates 1-4 were also tested to further characterize the performance of this enzymatic system. It was found that enzyme CYP109B1 showed relatively low coupling efficiency for all the substrate and the highest value of 11 was achieved for testosterone.

### Mechanism Study on CYP109B1 Catalyzed Steroid Hydroxylation

**FIGURE 3:**
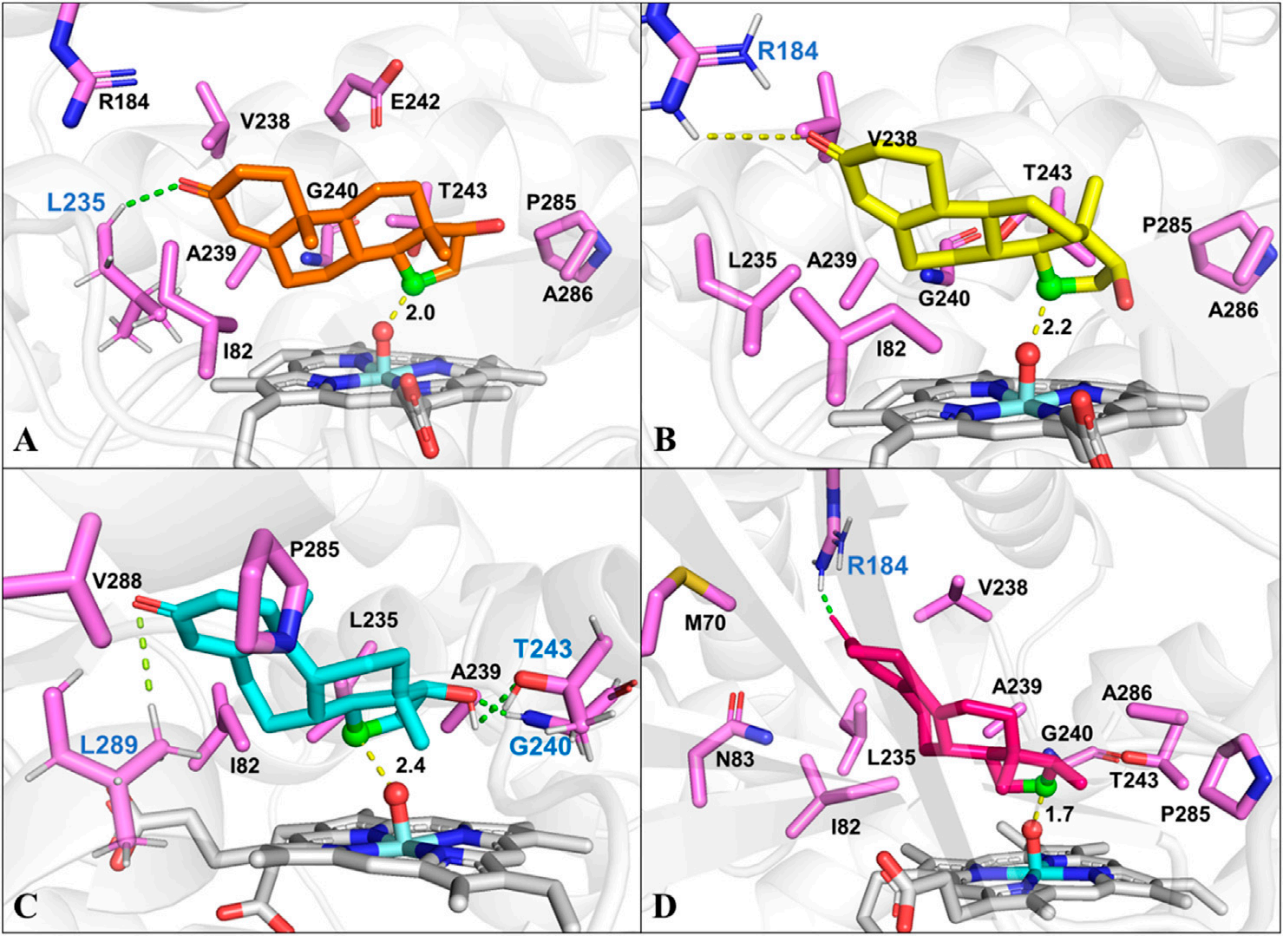
The docked conformation of steroid substrates in the active pocket of CYP109B1 (PDB ID: 4RM4). **(A)**: Testosterone. **(B)**: Nandrolone. **(C)**: Boldenone. **(D)**: 9 (10) dehydronandrolone .Key residues in active pocket of CYP109B1 are colored in violet and residues forming hydrogen bonds are labeled in blue. The important distance in the favorable docked poses are given in angstrom (Å). The C15 atom of steroid substrates are displayed in green with ball style and the Ox atom of Cpd I are displayed in red with ball style.

To identify the possible binding conformations of steroids in active site of CYP109B1, substrates 1-4 were docked into the active pocket of CYP109B1 (Supplementary Figures S19-S23). The docking results showed that the C15 β-hydrogen of testosterone (1) is well positioned for H-abstraction, with a distance of 2.0 Å between C-15 atom of 1 with the O atom of Cpd I (Figure 3A). In addition, hydrophobic interactions were formed between substrate 1 with residues Ile82, Leu235 and Ala239, Pro285 and Ala286 located in the active pocket of CYP109B1. For the analogous substrate nandrolone (2), the docked conformation is quite similar to that of 1, where C15 carbon atom of 2 maintains a distance of 2.2Å with the O atom of Cpd I (Figure 3B), leading to the formation of 15β-hydroxylated steroid as main product. Compared with substrates 1 and 2**,** the docked result of substrate boldenone (3) showed that additional H-bonding interactions were formed between C17-hydroxyl of 3 and Gly240 and Thr243, C3-keto of 3 and Leu289 (Figure 3C), which may increase the binding affinity and stability of 3. All these would in turn lead to the highest catalytic selectivity for substrate 3 among those steroids. As for substrate 9 (10)-dehydronandrolone (4)**,** different from the three steroids mentionsed above, C16 carbon atom of 4 keeps a short distance of 1.7 Å with the O atom of Cpd I. Along with the binding conformation of 4 (Figure 3D), the reaction would lead to the switched selectivity to produce the 16β-hydroxylated steroid.

**FIGURE 4:**
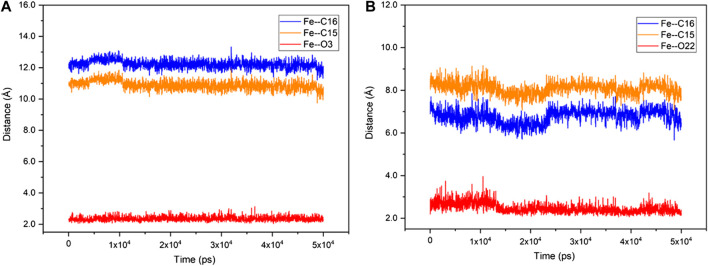
The fluctuation of the distance of Fe—O3 (red), Fe—C15 (orange) and Fe—C16 (blue) in CYP109B1-Progesterone systems **(A)** and CYP109B1-Progesterone systems **(B)** during the MD simulations.

Unlike the active steroid substrates 1-4, the substrates progesterone (5), canrenone (6), prednisolone (7) and pregnenolone (**8**) carrying the extra side chain at C17 were found to be inactive in experiments. To understand the root cause for this, the substrates 5 and 6 were further docked into the pocket of CYP109B1 in the resting state. As can be seen in Supplementary Figure S24, the O atom of keto at the A ring of substrate 5 and the E ring of substrate 6 was found to point toward the heme-iron center, with a short distance of 1.8 Å and 2.1 Å, respectively. Further MD simulations indicated that such binding conformations of substrate 5 and 6 are quite stable during 50 ns-MD simulation, during which the O atom of keto in substrate 5 and 6 maintains an average distance of 2.3 Å and 2.6 Å with the heme-iron, respectively. By contrast, the C15, C16 site in substrate 5 and 6 are quite far away from the heme-iron (Figure 4). As such, we speculate that stable electrostatic interactions between Fe and keto O atom may hinder the binding of initial O_2_ co-substrate, which in turn inhibit the formation of the active specie Cpd I. Thus, the extra side substitution at C17 of sterol seriously impedes the active binding of the O_2_ co-substrate which into active pocket of CYP109B1.

## Conclusion

In summary, we have established a more efficient CYP109B1 catalytic system for steroid hydroxylation by screening redox pairs from different resources or constructing the fused enzymes by fusing CYP109B1 to the N-terminal of reductase domain of self-sufficient P450 BM3 and P450RhF. The three-component system using the Fdr_0978/Fdx_1499 as redox partner showed the highest catalytic activity. Based on it, the substrate scope was tested for CYP109B1 and the subsequent computational analysis was performed , which enabled us to reveal the origin of regio- and stereoselective steroid hydroxylation of different steroids substrates. Future work will be focusing on the engineering of CYP109B1 to further improve the activity or selectivity for steroid hydroxylation, thus expanding its application in challenging biotransformation.

## Data Availability

The raw data supporting the conclusions of this article will be made available by the authors, without undue reservation.
