# Supplementary material for: Exploring the Potential of Cytochrome P450 CYP109B1 Catalyzed Regio—and Stereoselective Steroid Hydroxylation
Source: Front Chem. 2021 Feb 18;9:649000. doi: 10.3389/fchem.2021.649000 (PMC7930613; doi:10.3389/fchem.2021.649000)
Supplement: Supplementary file 1 [file table1.docx]

**Supporting information**

**Exploring the potential of cytochrome P450 CYP109B1 catalyzed regio- and stereoselective steroid hydroxylation**

Xiaodong Zhang,^1^^†^ Yun Hu,^1†^ Wei Peng,^2†^ Chenghua Gao,^1^ Qiong Xing,^1^* Binju Wang,^2^* Aitao Li^1^*

^1^State Key Laboratory of Biocatalysis and Enzyme Engineering, Hubei Collaborative Innovation Center for Green Transformation of Bio-Resources, Hubei Key Laboratory of Industrial Biotechnology, School of Life Sciences, Hubei University, Wuhan, 430062, P. R. China

^2^State Key Laboratory of Physical Chemistry of Solid Surfaces, Collaborative Innovation Center of Chemistry for Energy Materials, National Engineering Laboratory for Green Chemical Productions of Alcohols, Ethers and Esters, College of Chemistry and Chemical Engineering, Xiamen University

Xiamen 361005 (China)

*Email:[aitaoli@hubu.edu.cn;](mailto:aitaoli@hubu.edu.cn;%20) [qiongxingnmr@hubu.edu.cn](mailto:qiongxingnmr@hubu.edu.cn);

wangbinju2018@xmu.edu.cn

**Content**

[**NMR Characterization of the Products 3**](#_Toc60259869)

[**Tables 5**](#_Toc60259870)

[**Figures 9**](#_Toc60259871)

[**Nucleotide sequence information: 42**](#_Toc60259872)

[**Amino acid sequence information 53**](#_Toc60259873)

## NMR Characterization of the Products

**NMR data**

**15****β-hydroxy-testosterone (1a)** (16.0 mg, yield 27.7%)

^1^H NMR (400 MHz, dmso) δ 5.60 (d, J = 1.7 Hz, 1H), 4.46 (s, 1H), 4.33 (s, 1H), 3.93 (ddd, J = 8.1, 5.7, 2.6 Hz, 1H), 3.28 (t, J = 8.7 Hz, 1H), 2.36 (tdd, 1H), 2.30 – 2.18 (m, 2H), 2.17 – 2.01 (m, 2H), 1.99 – 1.89 (m, 1H), 1.80 (dt, 1H), 1.67 (dt, J = 12.5, 3.4 Hz, 1H), 1.56 (td, J = 14.1, 4.4 Hz, 1H), 1.50 – 1.25 (m, 3H), 1.13 (s, 3H), 0.87 (s, 3H), 0.95 – 0.79 (m, 4H), 0.66 – 0.60 (m, 1H).

^13^C NMR (101 MHz, dmso) δ 198.57, 171.89, 123.48, 80.09, 67.45, 55.29, 54.27, 43.51, 42.22, 38.09, 35.65, 34.07, 32.54, 31.48, 31.07, 20.67, 17.33, 14.12.

**15β-hydroxy-nandrolone (2a)** (69.6 mg, yield 25.4%)

^1^H NMR (400 MHz, dmso) δ 5.69 (s, 1H), 4.46 (d, J = 4.9 Hz, 1H), 4.32 (d, J = 4.0 Hz, 1H), 3.94 (qd, J = 4.9 Hz, 1H), 3.29 (dt, J = 8.6, 4.6 Hz, 1H), 2.39 (dt, 1H), 2.29 (ddd, 1H), 2.25 (d, J = 7.6 Hz, 1H), 2.19 (d, J = 6.9 Hz, 3H), 2.15 (d, J = 3.0 Hz, 1H), 2.08 (dp, J = 10.7, 2.5 Hz, 1H), 1.75 – 1.65 (m, 1H), 1.63 (dt, J = 3.2, 2.7 Hz, 1H), 1.58 (qd, J = 11.1, 2.8 Hz, 1H), 1.39 (qd, J = 8.8, 2.7 Hz, 2H), 1.18 (td, 2H), 1.02 – 0.85 (m, 2H), 0.90 (s, 4H), 0.77 (td, 1H), 0.66 (dd, J = 11.3, 5.7 Hz, 1H).

^13^C NMR (101 MHz, dmso) δ 199.00, 167.81, 124.05, 80.17, 67.45, 54.59, 49.97, 43.57, 42.45, 38.13, 36.63, 36.41, 35.19, 30.15, 26.64, 26.02, 14.10.

**16β-hydroxy-nandrolone (2b)** (37.2 mg, yield 13.5%)

^1^H NMR (400 MHz, dmso) δ 5.70 (d, J = 1.8 Hz, 1H), 4.50 (d, J = 3.9 Hz, 1H), 4.09 (d, J = 7.2 Hz, 1H), 3.90 (dq, J = 8.0, 3.9 Hz, 1H), 3.15 (t, J = 7.3 Hz, 1H), 2.45 – 2.35 (m, 1H), 2.26 (dd, J = 13.9, 5.1 Hz, 1H), 2.22 – 2.12 (m, 4H), 2.03 (dt, J = 12.8, 7.4 Hz, 1H), 2.31 – 1.96 (m, 0H), 1.79 – 1.71 (m, 2H), 1.67 (dt, J = 4.4, 3.2 Hz, 0H), 1.48 – 1.30 (m, 2H), 1.30 – 1.17 (m, 5H), 1.16 – 1.01 (m, 1H), 0.80 (qd, J = 10.2 Hz, 0H), 0.74 (s, 3H).

^13^C NMR (101 MHz, dmso) δ 198.83, 167.21, 124.18, 80.38, 69.00, 49.71, 46.24, 42.56, 42.22, 39.62, 37.35, 36.61, 35.23, 35.08, 30.98, 26.56, 25.85, 12.60.

**15β-hydroxy-boldenone (3a)** (189.8 mg, yield 66.3%)

^1^H NMR (400 MHz, dmso) δ 7.18 (d, *J* = 10.1 Hz, 1H), 6.08 (d, *J* = 10.1 Hz, 1H), 5.95 (s, 1H), 4.48 (d, *J* = 4.9 Hz, 1H), 4.39 (d, *J* = 3.9 Hz, 1H), 3.91 (dt, *J* = 4.6, 4.1 Hz, 1H), 3.27 (td, *J* = 8.6, 4.6 Hz, 1H), 2.42 (td, 1H), 2.26 (dd, *J* = 70.3, 16.8 Hz, 3H), 1.92 (qd, *J* = 11.2, 3.4 Hz, 1H), 1.67 (td, 1H), 1.60 (qd, 2H), 1.39 (ddd, *J* = 14.1, 8.8, 2.5 Hz, 1H), 1.18 (s, 3H), 0.91 (s, 7H), 0.62 (dd, *J* = 11.1, 5.6 Hz, 1H).

^13^C NMR (101 MHz, dmso) δ 185.52, 170.39, 157.19, 127.09, 123.33, 79.98, 67.63, 54.79, 53.23, 43.93, 43.48, 42.53, 37.98, 32.63, 32.53, 31.66, 22.55, 18.94, 14.20.

**15β-hydroxy-9(10) dehydronandrolone (4a)** (92.6 mg, yield 16.2%)

^1^H NMR (400 MHz, dmso) δ 5.53 (s, 1H), 4.68 – 4.34 (m, 4H), 3.94 (td, 1H), 3.30 (t, 3H), 2.84 (dt, J = 14.9, 5.4 Hz, 1H), 2.72 (dd, 1H), 2.58 – 2.39 (m, 2H), 2.34 (dd, J = 6.4, 4.0 Hz, 2H), 2.33 – 2.24 (m, 4H), 2.13 (td, 1H), 2.02 (td, 1H), 1.73 (ddd, J = 12.5, 5.5, 2.2 Hz, 1H), 1.45 (td, J = 8.9, 2.5 Hz, 2H), 1.15 (t, J = 7.1 Hz, 1H), 1.04 (t, J = 4.4 Hz, 0H), 0.99 (s, 6H), 0.80 (ddd, J = 12.3, 6.4, 6.4 Hz, 2H).

^13^C NMR (101 MHz, dmso) δ 198.36, 157.41, 148.24, 125.27, 121.62, 79.75, 67.27, 55.68, 43.76, 42.26, 38.18, 37.19, 35.58, 30.68, 26.57, 25.73, 25.68, 13.42.

**16β-hydroxy-9(10) dehydronandrolone (4b)** (223.6 mg, yield 41.0%)

^1^H NMR (400 MHz, cdcl3) δ 5.67 (s, 1H), 4.20 (td, J = 7.7, 4.8 Hz, 1H), 3.40 (d, J = 7.5 Hz, 1H), 2.88 (dt, J = 14.3, 5.0 Hz, 1H), 2.79 (ddd, J = 16.1, 4.8, 2.2 Hz, 1H), 2.60 – 2.46 (m, 1H), 2.46 – 2.37 (m, 4H), 2.37 – 2.21 (m, 3H), 2.19 – 2.04 (m, 1H), 2.00 – 1.85 (m, 2H), 1.36 (td, J = 13.3, 4.8 Hz, 2H), 1.31 – 1.20 (m, 3H), 0.96 (s, 4H).

^13^C NMR (101 MHz, cdcl3) δ 199.90, 157.22, 146.13, 125.64, 122.17, 80.44, 70.00, 47.62, 42.39, 38.75, 37.13, 37.02, 35.04, 30.77, 27.29, 25.76, 25.37, 11.27.

# Tables

**Table S1.** List of primers used in this study.

| **Primer cloning** | **Primer sequence (5’→3’)** |
| --- | --- |
| **pRSFDute-1-F** | GCGGCCGCATAATGCTTAAG |
| **pRSFDute-1-R** | AAGCTTGTCGACCTGCAGGC |
| **CYP109B1-F** | ATGAATGTGTTAAACCGCCGGC |
| **CYP109B1-R** | TTACATTTTCACACGGAAGCTCTTTAATCCG |
| **pRSF-CYP109B1(SOE)-F** | CAGGTCGACAAGCTTATGAATGTGTTAAACCGCCGGC |
| **pRSF-CYP109B1(SOE)-R** | GCATTATGCGGCCGCTTACATTTTCACACGGAAGCTCTTTAATCCG |
| **Fdr-F** | ATGTTGAATGCGAGTGTGGCTG |
| **Fdr-R** | CTAGTAGGTTTCAACATGCCAACGACC |
| **Fdr(SOE)-F** | CAGGTCGACAAGCTTATGTTGAATGCGAGTGTGGCTG |
| **Fdr(SOE)-R** | GCATTATGCGGCCGCCTAGTAGGTTTCAACATGCCAACGACC |
| **Fdx-F** | ATGGCAACCTACAAGGTTACGCT |
| **Fdx-R** | CTAGTAGAGGTCTTCTTCTTTGTGGGTTTCG |
| **Fdx(SOE)-F** | TCGACAAGCTTATGGCAACCTACAAGGTTACGCTC |
| **Fdx(SOE)-R** | GCATTATGCGGCCGCTTAGTAGAGGTCTTCTTCTTTGTGGGTTTCG |
| **Fdr(RBS)-F** | TCTCAATTGGATATCATGTTGAATGCGAGTGTGGCTG |
| **Fdr(RBS)-R** | GATATATCTCCTTAGGTACCCTAGTAGGTTTCAACATGCCAACGACC |
| **Fdx(RBS)-F** | GGTACCTAAGGAGATATATCATGGCAACCTACAAGGTTACGCT |
| **Fdx(RBS)-F** | ATCGCGTGGCCGGCCCTAGTAGAGGTCTTCTTCTTTGTGGGTTTCG |
| **FNR-F** | ATGCAGATCGCCTCTGATGTGG |
| **FNR-R** | TTAGTAGACTTCAACGTTCCATTGTTCTGCC |
| **FNR(SOE)-F** | CAGGTCGACAAGCTTATGCAGATCGCCTCTGATGTG |
| **FNR(SOE)-R** | GCATTATGCGGCCGCTTAGTAGACTTCAACGTTCCATTGTTCTGCC |
| **Fd I-F** | ATGGCCGCCTACAAGGTGAC |
| **Fd I-R** | TTAGGCGGTCAGCTCCTCTTCTTTG |
| **Fd I(SOE)-F** | CAGGTCGACAAGCTTATGGCCGCCTACAAGGTGAC |
| **Fd I(SOE)-R** | GCATTATGCGGCCGCTTAGGCGGTCAGCTCCTCTTCTTTG |
| **FNR(RBS)-F** | TCTCAATTGGATATCATGCAGATCGCCTCTGATGTGG |
| **FNR(RBS)-R** | GATATATCTCCTTAGGTACCTTAGTAGACTTCAACGTTCCATTGTTCTGCC |
| **Fd I(RBS)-F** | GGTACCTAAGGAGATATATCATGGCCGCCTACAAGGTGAC |
| **Fd I(RBS)-R** | ATCGCGTGGCCGGCCTTAGGCGGTCAGCTCCTCTTCTT |
| **CYP109B1-RhF-R** | ATGCCGGTGCAGCACTTACATTTTCACACGGAAGCTCTTTAATCCG |
| **RhF-F** | GTGCTGCACCGGCATCAAC |
| **RhF(SOE)-R** | GCATTATGCGGCCGCTCAGAGTCGCAGGGCCAGC |
| **CYP109B1-BM3-R** | AGTGCTAGGTGAAGGTTACATTTTCACACGGAAGCTCTTTAATCCG |
| **BM3-F** | CCTTCACCTAGCACTGAACAGTCTGC |
| **BM3(SOE)-R** | GCATTATGCGGCCGCTTACCCAGCCCACACGTCTTTTGC |
| **Fpr-F** | ATGGCTGATTGGGTAACAGGCAAAG |
| **Fpr-R** | TTACCAGTAATGCTCCGCTGTCATATG |
| **Fpr(SOE)-F** | CAGGTCGACAAGCTTATGGCTGATTGGGTAACAGGCAAAG |
| **Fpr(SOE)-R** | GCATTATGCGGCCGCTTACCAGTAATGCTCCGCTGTCATATGG |
| **YkuN-F** | ATGGCTAAAGCCTTGATTACATATGCCAG |
| **YkuN-R** | TTATGAAACATGGATTTTTTCCTTGTTCATATAATC |
| **YkuN(SOE)-F** | CAGGTCGACAAGCTTATGGCTAAAGCCTTGATTACATATGCCAG |
| **YkuN(SOE)-R** | GCATTATGCGGCCGCTTATGAAACATGGATTTTTTCCTTGTTCATATAATC |
| **YkuP-F** | ATGGCGAAGATTTTGCTCGTTTATGC |
| **YkuP -R** | TCAGACAGCGCACCCGC |
| **YkuP (SOE)-F** | CAGGTCGACAAGCTTATGGCGAAGATTTTGCTCGTTTATGC |
| **YkuP (SOE)-R** | GCATTATGCGGCCGCTCAGACAGCGCACCCGC |
| **Fpr(RBS)-F** | TCTCAATTGGATATCATGGCTGATTGGGTAACAGGCAAAG |
| **Fpr(RBS)-R** | GATATATCTCCTTAGGTACCTTACCAGTAATGCTCCGCTGTCATATG |
| **YkuN(RBS)-F** | GGTACCTAAGGAGATATATCATGGCTAAAGCCTTGATTACATATGCCAG |
| **YkuN(RBS)-R** | ATCGCGTGGCCGGCCTTATGAAACATGGATTTTTTCCTTGTTCATATAATC |
| **YkuP(RBS)-F** | GGTACCTAAGGAGATATATCATGGCGAAGATTTTGCTCGTTTATGC |
| **YkuP(RBS)-R** | ATCGCGTGGCCGGCCTCAGACAGCGCACCCGC |

**Table S2**. Determination of the concentration of CYP109B1.

| **Group** | **A_450nm_** | **A_490nm_** | **A_450nm_-A_490nm_** | **C(CYP109B1) (μM)** |
| --- | --- | --- | --- | --- |
| **A1** | 0.2635 | 0.1172 | 0.1463 | ‾A(450nm-490nm) ×100^a^/0.091^b^ mM^-1^ cm^-1^ |
| **A2** | 0.1886 | 0.0245 | 0.1641 |  |
| **A3** | 0.2247 | 0.0702 | 0.1545 |  |
| **B1** | 0.1762 | 0.0129 | 0.1633 |  |
| **B2** | 0.2384 | 0.0761 | 0.1623 |  |
| **B3** | 0.2933 | 0.1334 | 0.1599 |  |

^a^ Diluted multiples pure enzyme of CYP109B1.

^b^ Extinction coefficient

**Table S3**. The conditions for HPLC analysis. ^a^

| **Compounds** | **Retention time(min)** | **Mobile phase (%)** ^b^ |
| --- | --- | --- |
| Testosterone | 9.061 | 0 → 1 min: A: B: C = 70:15:15  1 → 6 min: A: B: C = 30:35:35  6 → 9 min: A: B: C = 70:15:15  9 → 10 min: A: B: C = 70:15:15 |
| Nandrolone | 8.641 |  |
| Adrenosterone | 7.361 |  |
| Boldenone | 10.456 | 0 → 1 min: A: B: C = 70:15:15  1 → 2 min: A: B: C = 60:20:20  2 → 17 min: A: B: C = 20:40:40  17 → 20 min: A: B: C = 70:15:15 |
| 9(10)-Dehydronandrolone | 10.157 |  |
| Canreone | 13.180 |  |
| β-Estradiol | 11.004 |  |
| Prednisolone | 7.449 |  |
| Pregnenolone | 13.024 | 0 → 1 min: A: C = 85:15  1 → 15 min: A: C = 10:90  15 → 18 min: A: C = 85:15  18 → 20 min: A: C = 85:15 |
| Progesterone | 16.958 | 0 → 1 min: A: B = 70:30  1 → 15 min: A: B = 20:80  15 → 18 min: A: B = 70:30  18 → 20 min: A: B = 70:30 |

^a^ Retention time of the substrate peaks out.

^b^ Mobile phase is changing over time, **A**: ultra-pure water was processed with filtration and ultrasonic degassing, **B**: Chromatographic grade methanol was processed with filtration and ultrasonic degassing, **C**: Chromatographic grade acetonitrile was processed with filtration and ultrasonic degassing.

# Figures

**
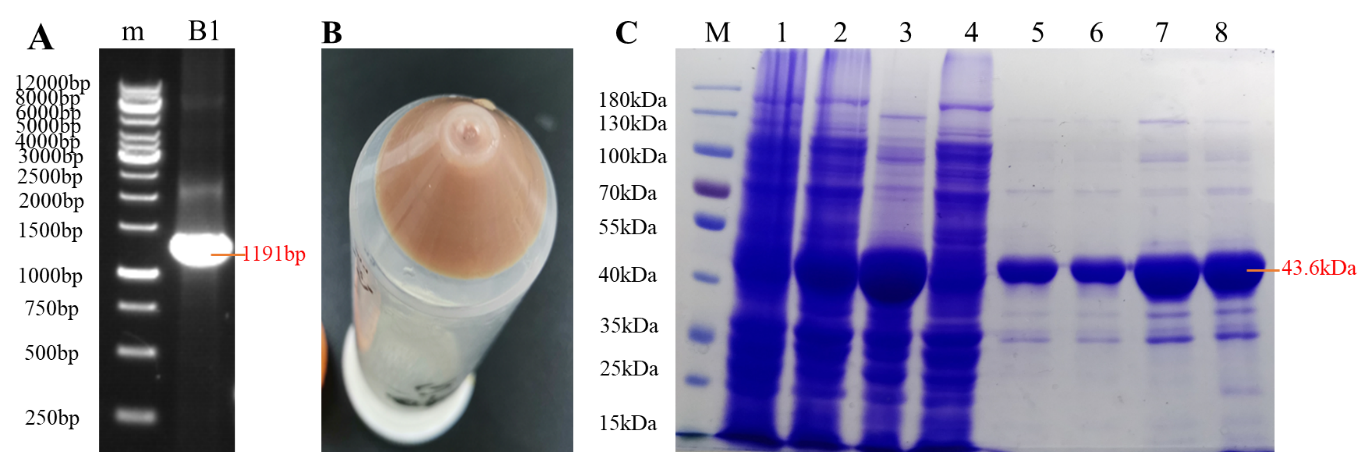
**

**Figure S1.** The nucleic acid gel (**A**), cell of CYP109B1 (**B**) and SDS-PAGE gel (**C**) of the purification of CYP109B1.

Line m: DNA standard marker, line B1: nucleotide fragment of CYP109B1.

Line M: protein standard marker, line 1: crude enzyme, line 2: supernatant of bacterial breaking fluid, line 3: sediment of bacterial breaking fluid, line 4: flowed through, line 5 to 8: eluant of 10 mM, 20 mM, 40 mM and 250 mM imidazole. The theoretical protein size of CYP109B1 is 43.6 kDa (marked in red).


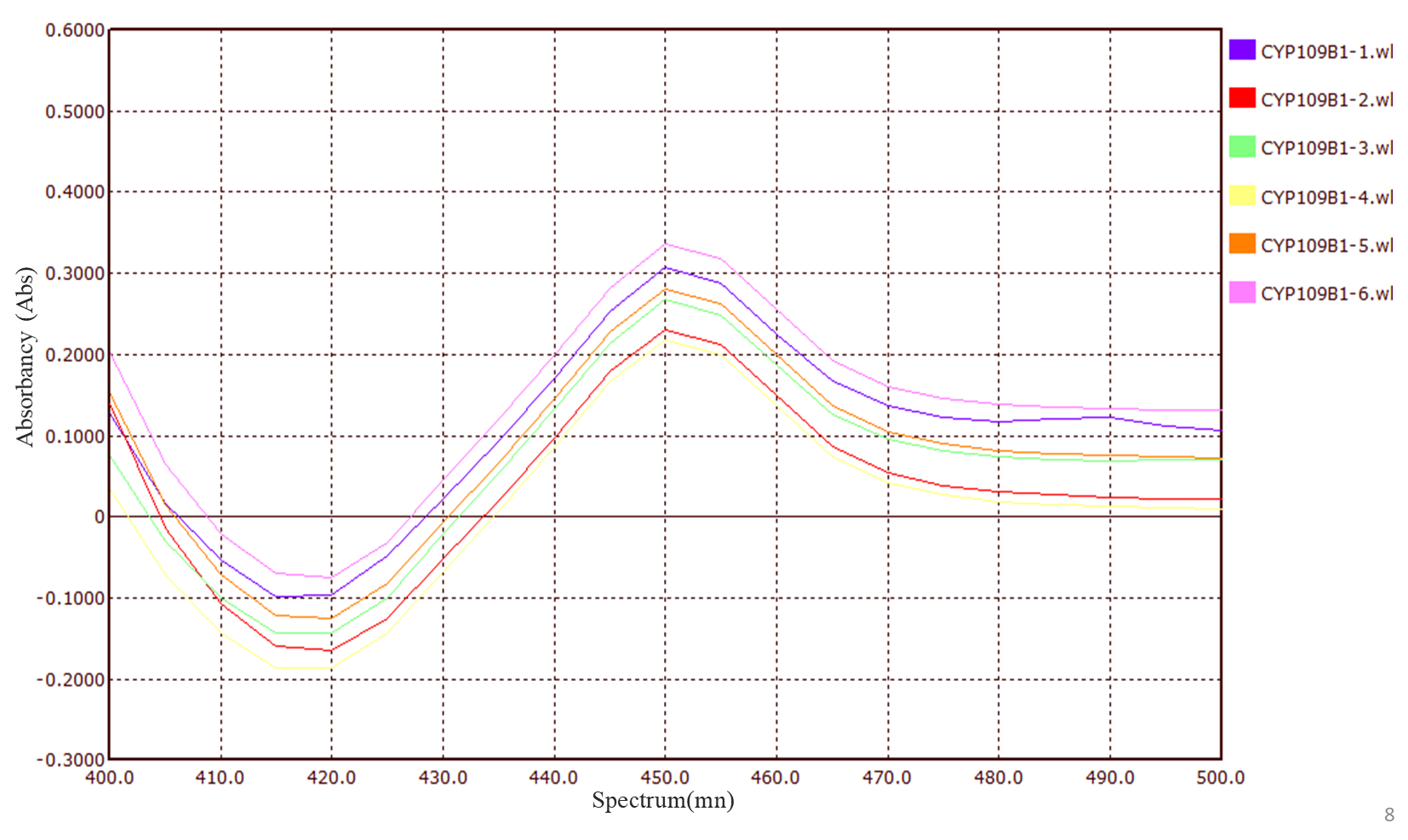


**Figure S2**. The CO spectral characterization and determination of protein concentration for purifiedCYP109B1 based on 100 times dilution.


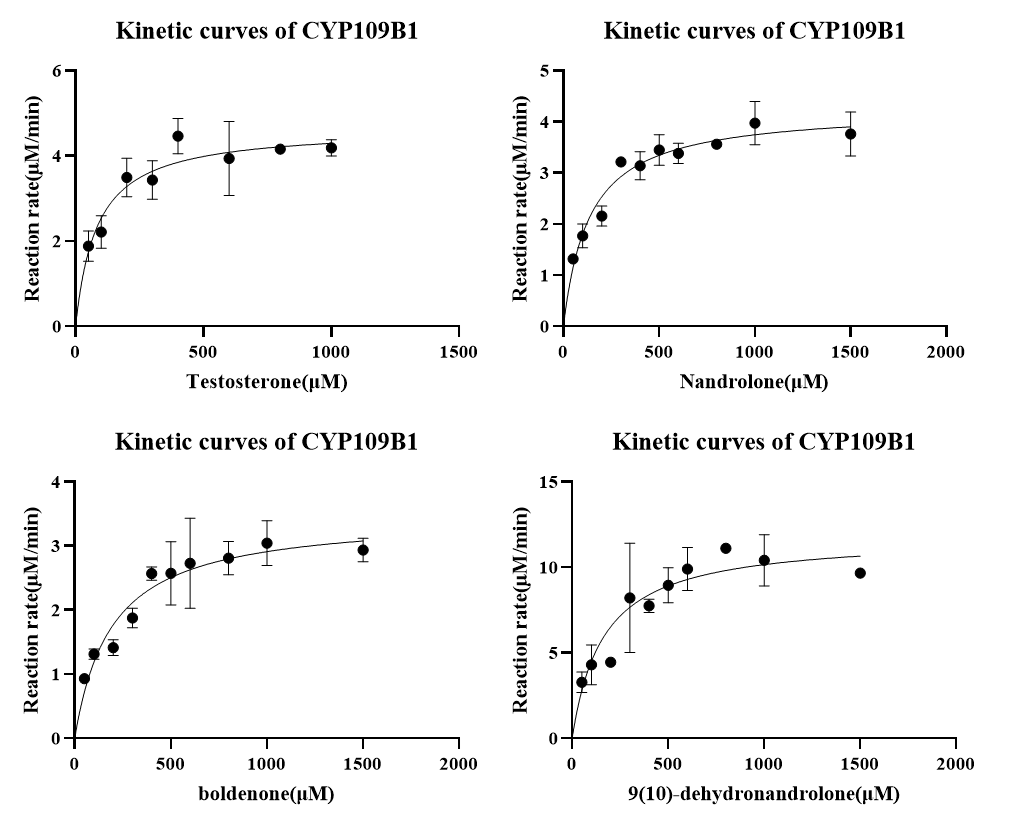


**Figure S3**. Steady-state kinetic behavior of evolved CYP109B1. Conditions: 1 μM mixture enzyme, steroids (0-1.5 mM), NADPH regeneration system (5 g/L glucose-6-phosphate, 5 g/L glycerol, 1 unit glucose-6-phosphate dehydrogenase and 1 mM NADPH). Reactions were allowed to proceed in 5 minutes with shaking at 750 rpm at 30 ℃.


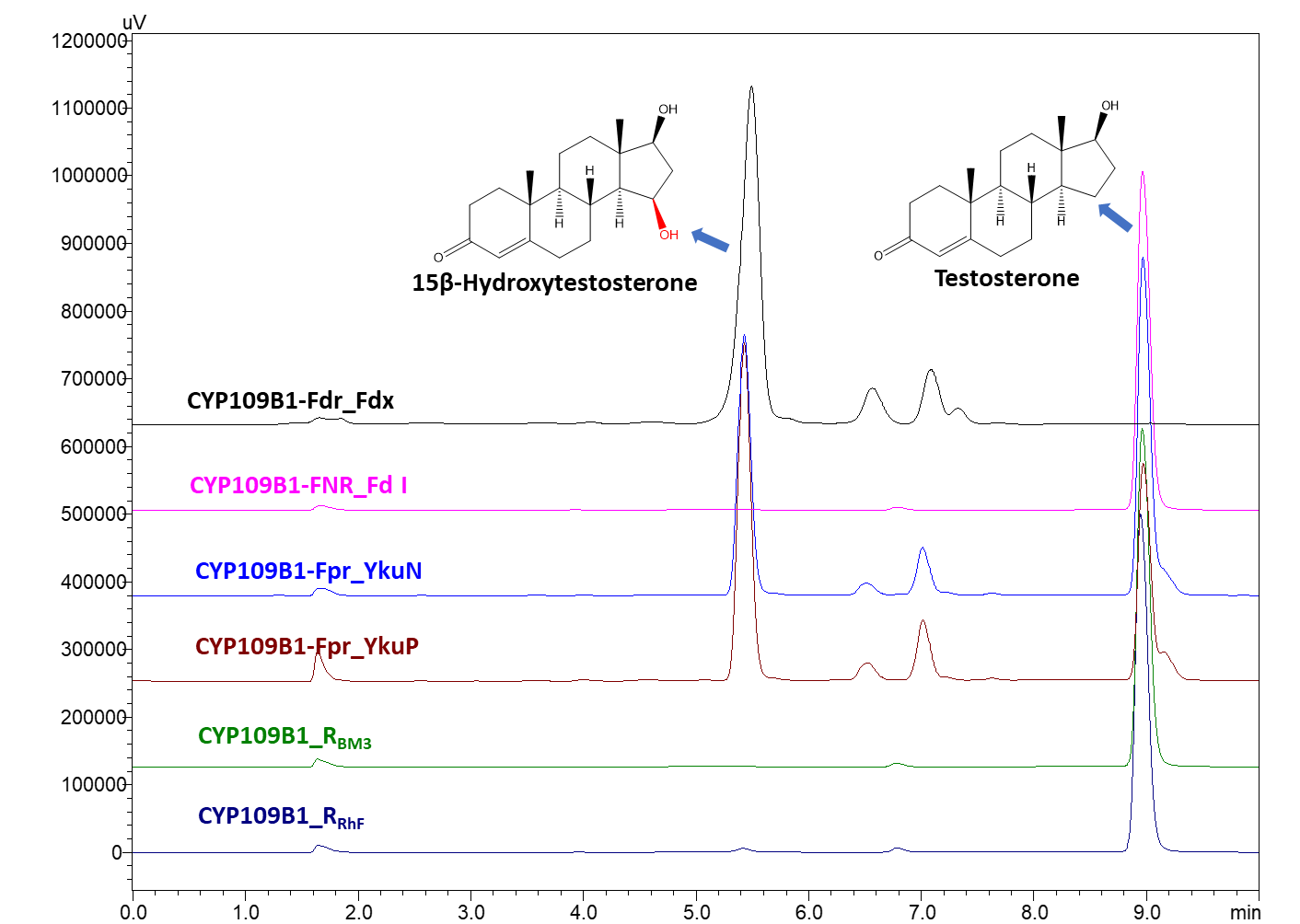


**Figure S4**. HPLC chromatograms of regio- and stereoselective 15β-hydroxylation of testosterone (**1**) catalyzed by CYP109B1with different redox partners. The flavodoxin reductase (Fpr) from *Escherichia coli*, flavodoxins (YkuN or YkuP) from *B. subtilis*, ferredoxin reductase (Fdr_0978) and ferredoxin (Fdx_1499) from *Synechococcus elongates* PCC7942, ferredoxin reductase (FNR) and ferredoxin (Fd I) from *spinach*, R_BM3_ reductase region of P450BM3 from *B. megatherium* and R_RhF_ reductase region of P450RhF from *Rhodococcus sp.* Strain NCIMB 9784.


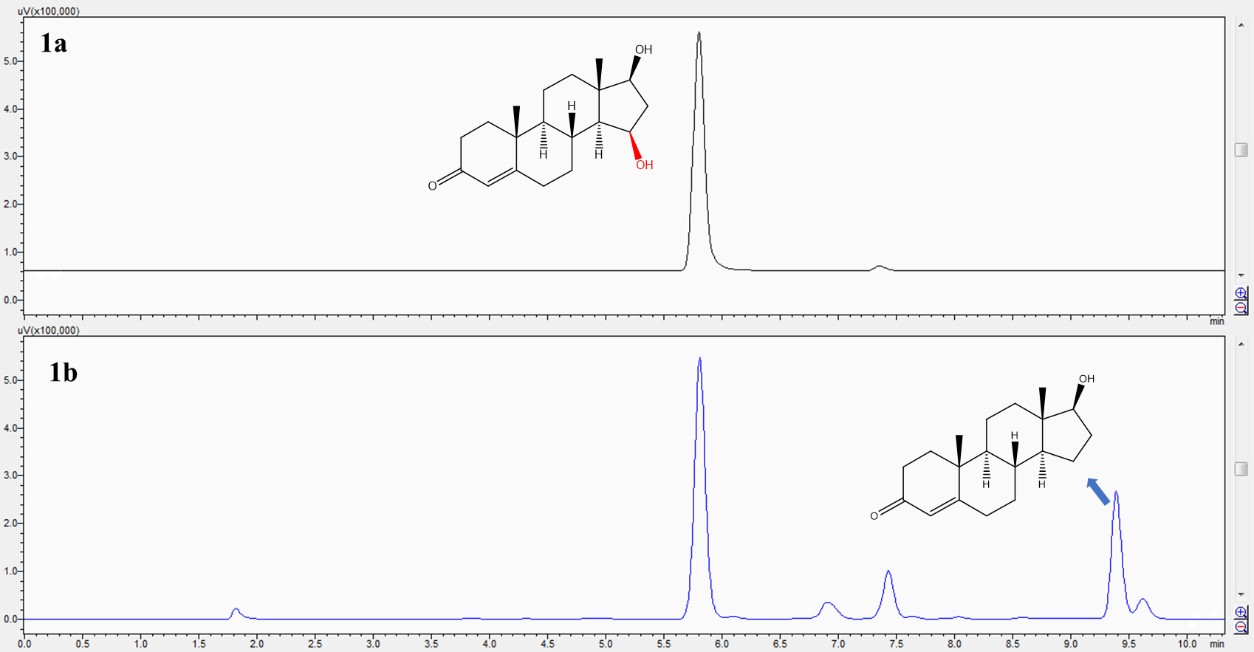


**Figure S5**. HPLC chromatograms of regio- and stereoselective 15β-hydroxylation of testosterone (**1**) catalyzed by CYP109B1: (**1a**) product after purification and (**1b**) reaction mixture.


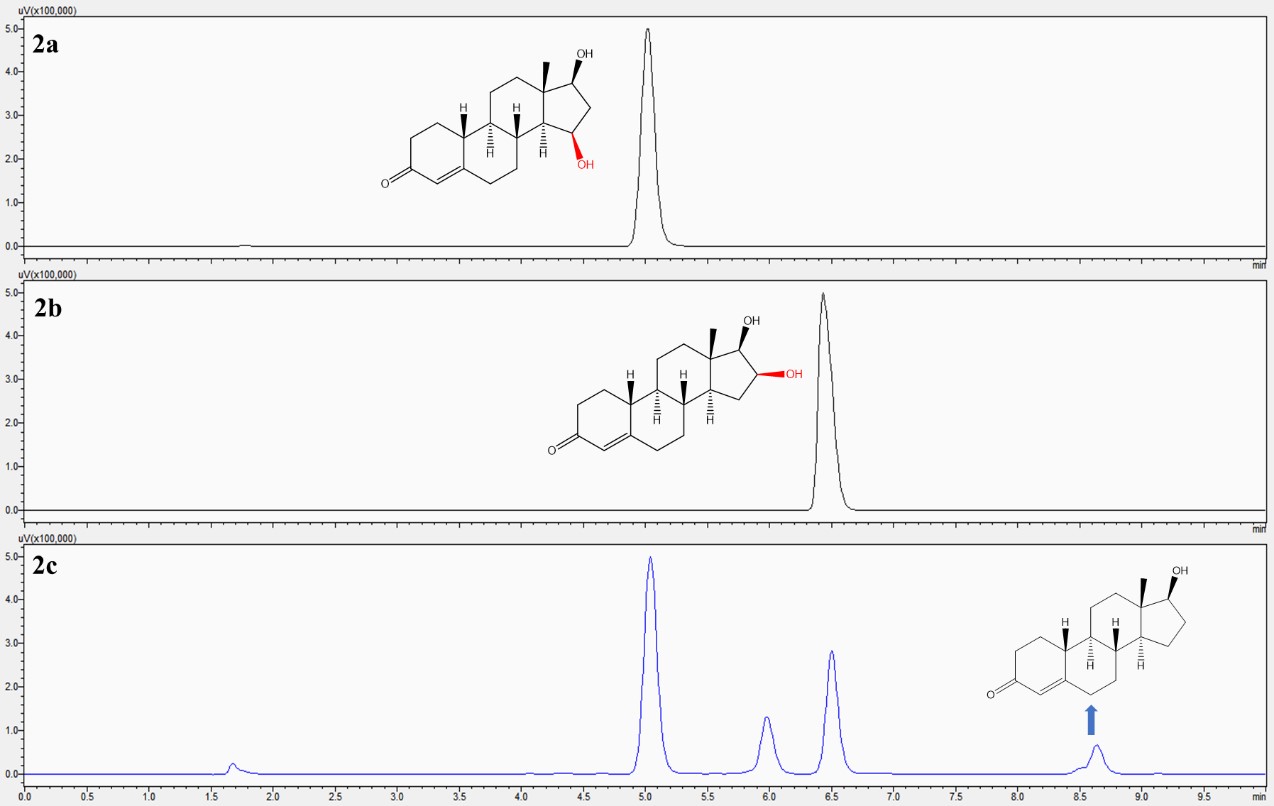


**Figure S6**. HPLC chromatograms of regio- and stereoselective 15β- and 16β-hydroxylation of nandrolone (**2**) catalyzed by CYP109B1: (**2a**, **2b**) products after purification and (**2c**) reaction mixture.


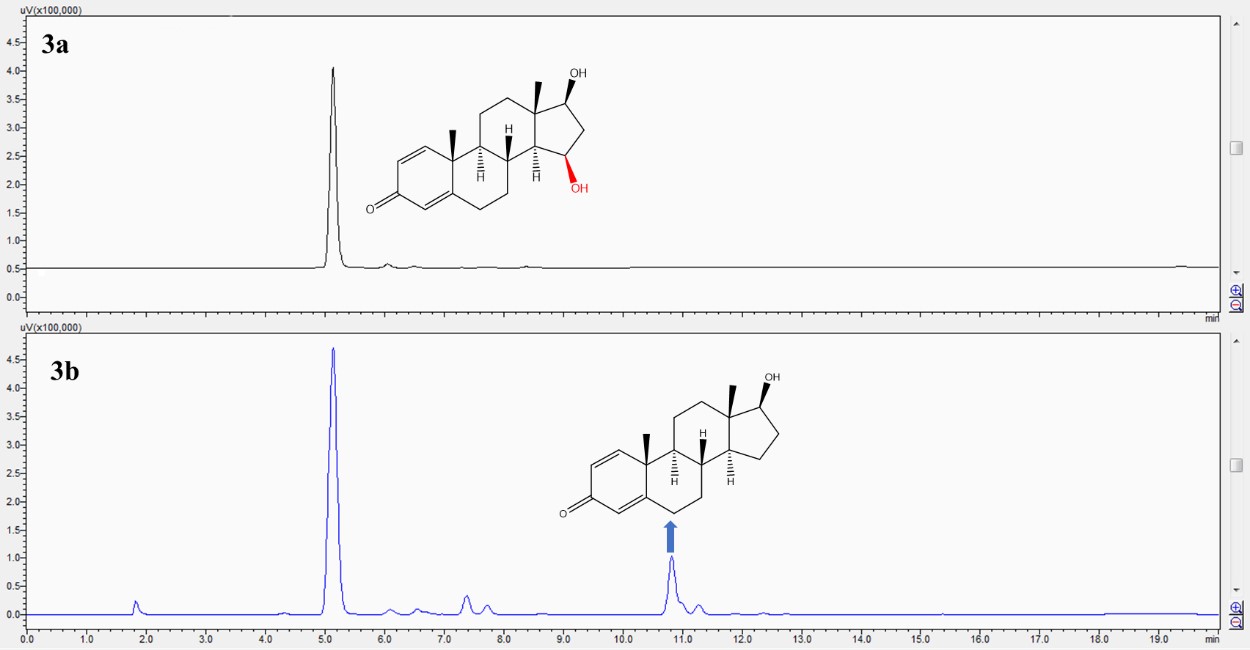


**Figure S7**. HPLC chromatograms of regio- and stereoselective 15β-hydroxylation of boldenone (**3**) catalyzed by CYP109B1: (**3a**) products after purification and (**3b**) reaction mixture.


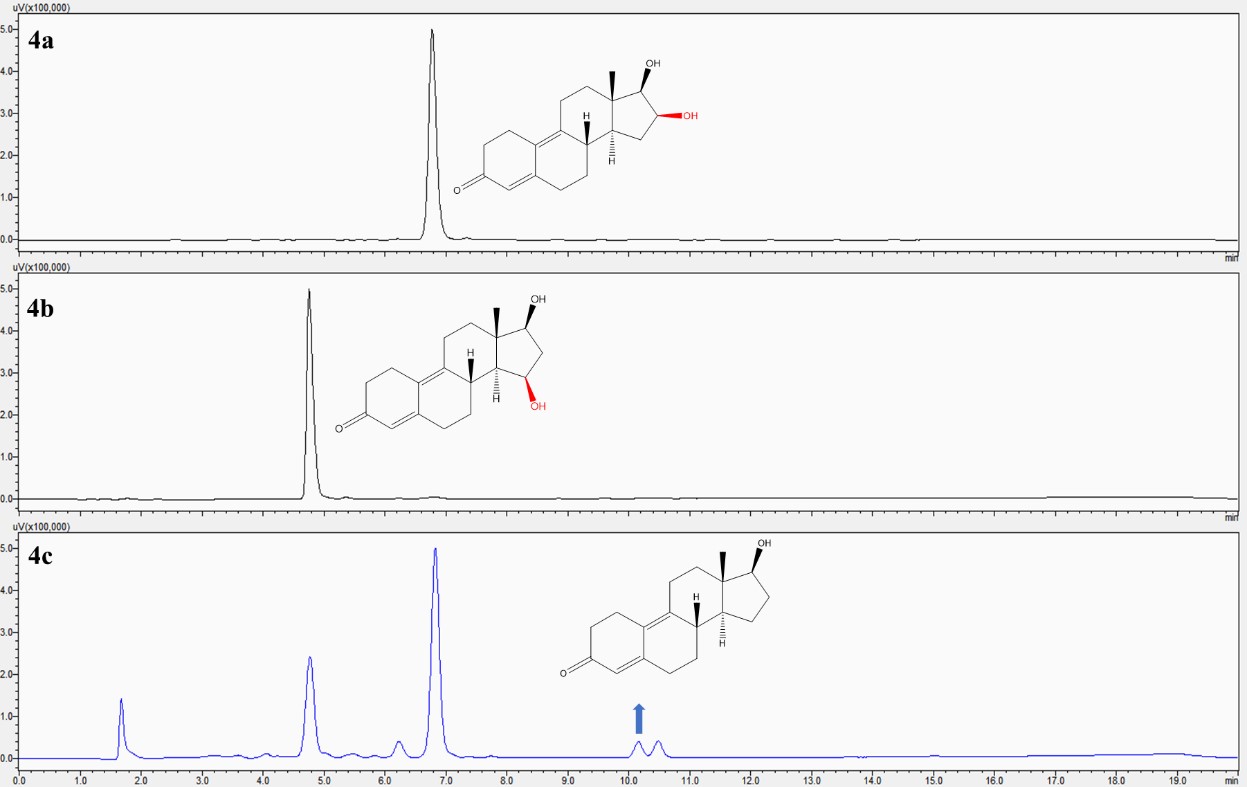


**Figure S8**. HPLC chromatograms of regio- and stereoselective 15β- and 16β-hydroxylation of 9(10)-dehydronandrolone (**4**) catalyzed by CYP109B1: (**4a, 4b**) products after purification and (**4c**) reaction mixture.


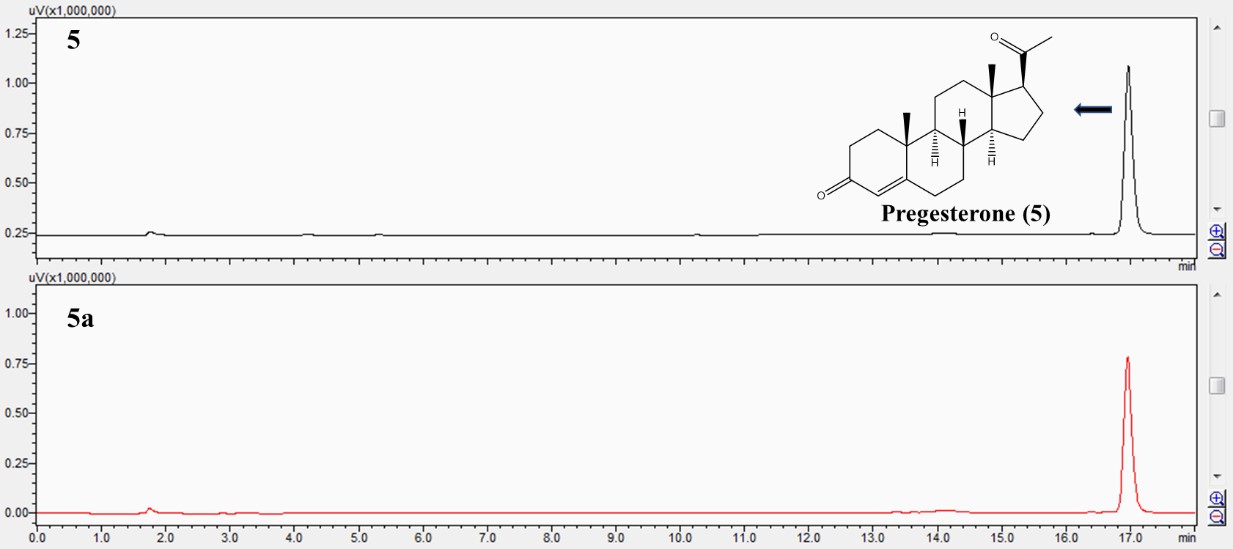


**Figure S9**. HPLC chromatograms of progesterone (**5**) and reaction mixture (**5a**) catalyzed by CYP109B1.


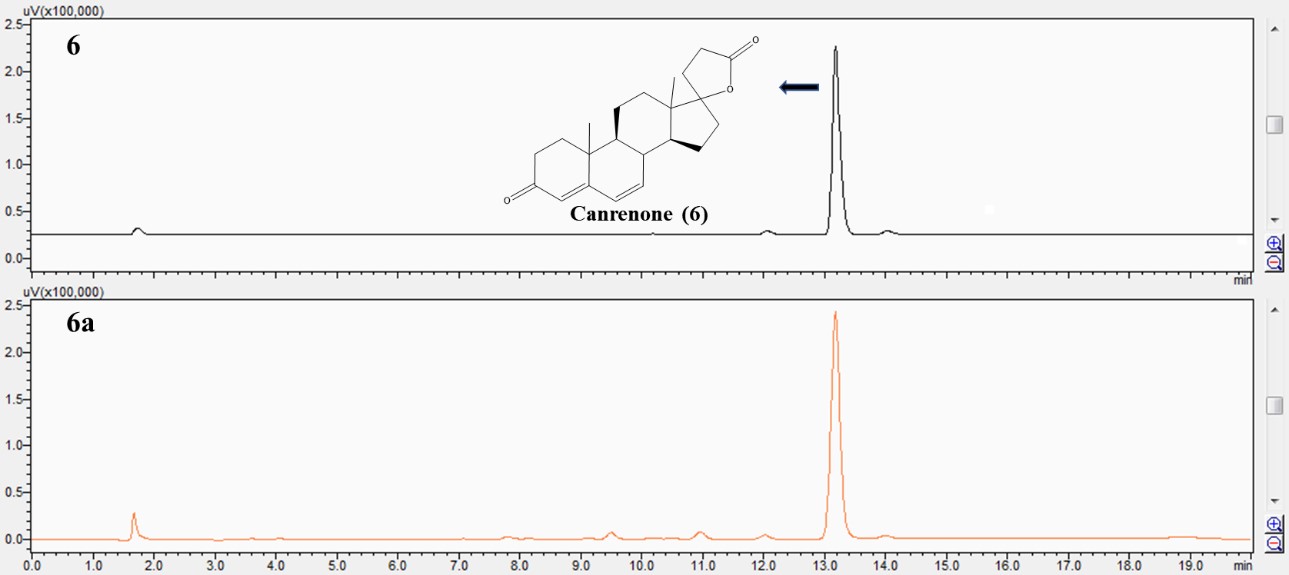


**Figure S10**. HPLC chromatograms of canrenone (**6**) and reaction mixture (**6a**) catalyzed by CYP109B1.


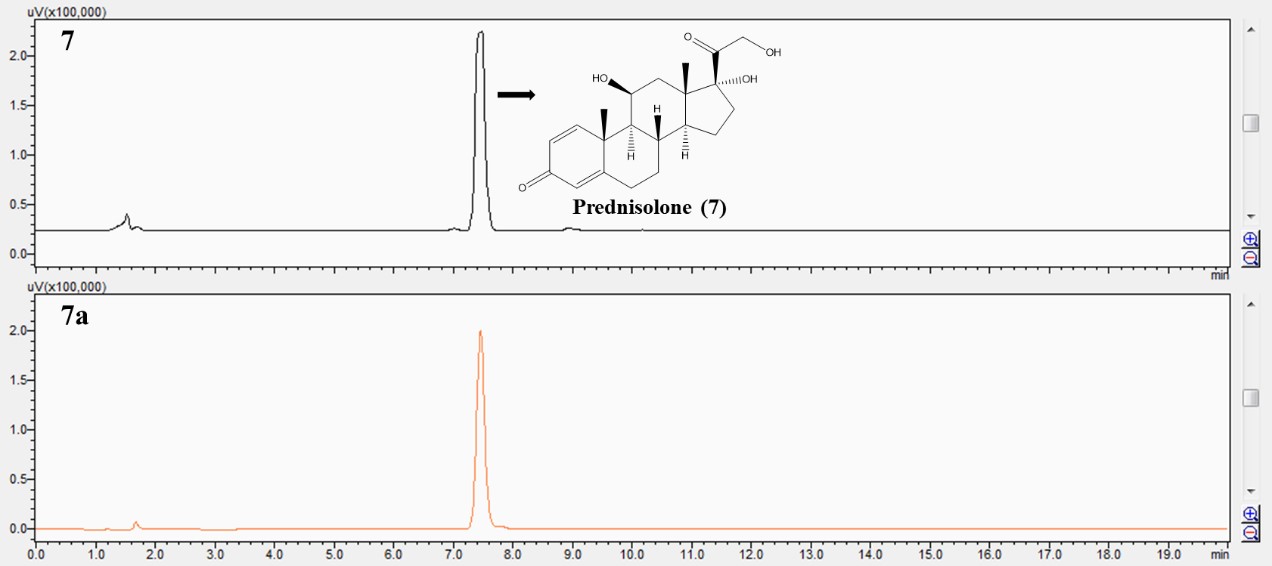


**Figure S11**. HPLC chromatograms of prednisolone (**7**) and reaction mixture (**7a**) catalyzed by CYP109B1.


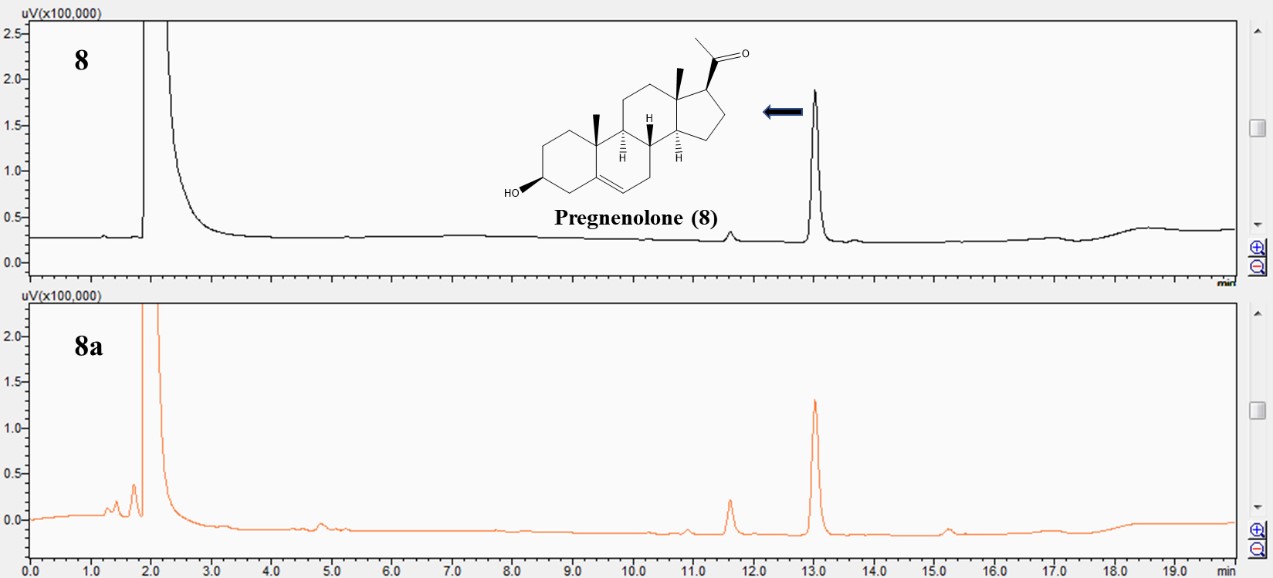


**Figure S12**. HPLC chromatograms of pregnenolone (**8**) and reaction mixture (**8a**) catalyzed by CYP109B1.


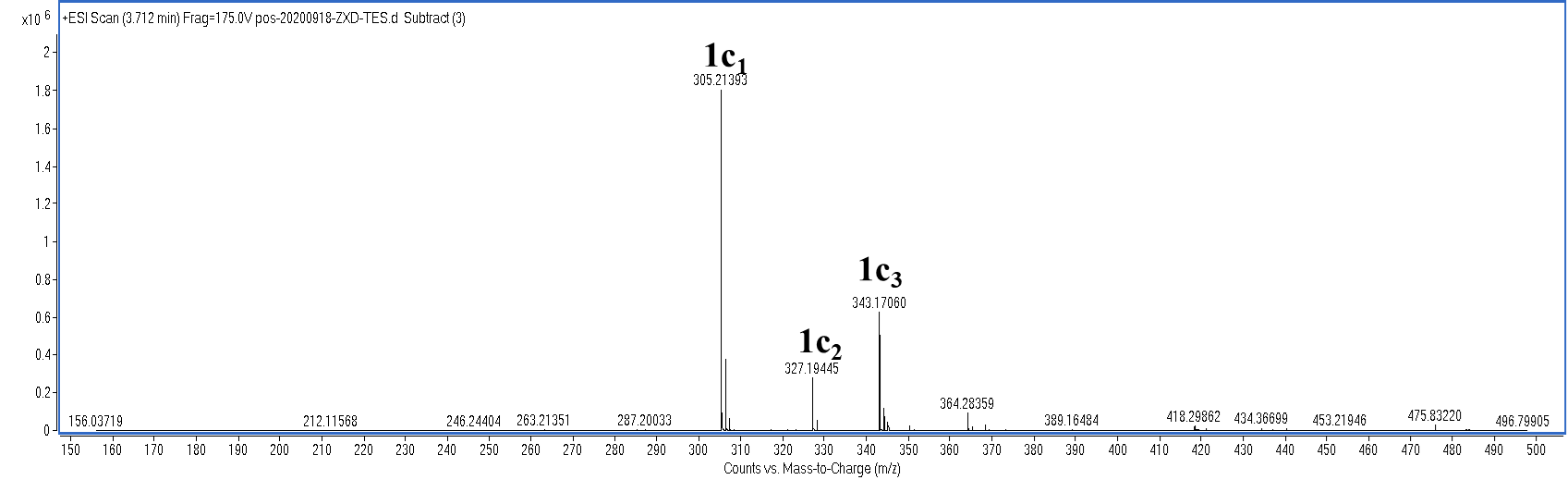


**Figure S13**. Mass Spectrum analysis for 15β-hydroxytestosterone (**1a**). Protonated peak (**1c_1_**), ionized with sodium ion peak (**1c_2_**) and ionized with potassium ion peak (**1c_3_**) of 15β-hydroxytestosterone.


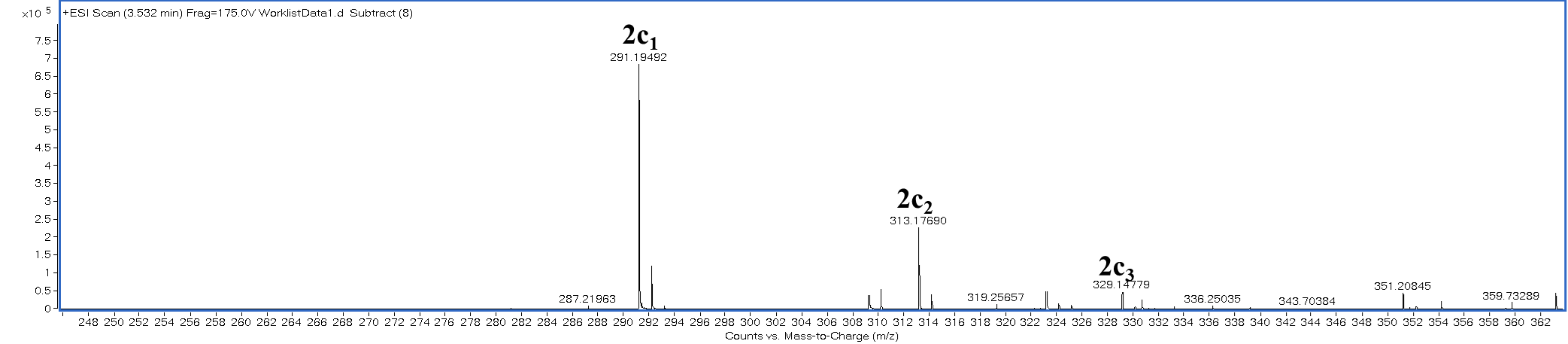


**Figure S14**. Mass Spectrum analysis for 15β-hydroxynandrolone (**2a**). Protonated peak (**2c_1_**), ionized with sodium ion peak (**2c_2_**) and ionized with potassium ion peak (**2c_3_**) of 15β-hydroxynandrolone.


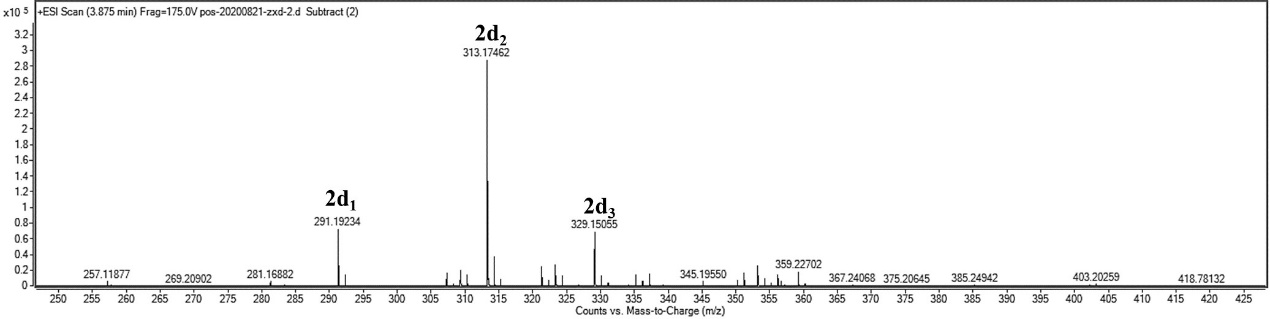


**Figure S15**. Mass Spectrum analysis for 16β-hydroxynandrolone (**2a**). Protonated peak (**2d_1_**), ionized with sodium ion peak (**2d_2_**) and ionized with potassium ion peak (**2d_3_**) of 16β-hydroxynandrolone.


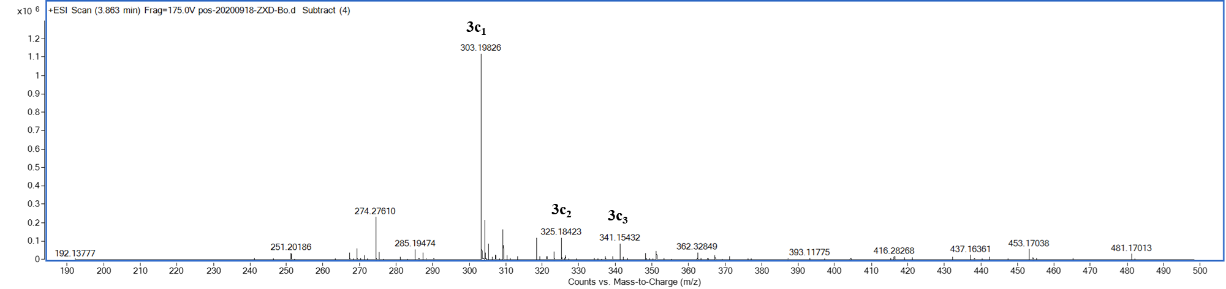


**Figure S16**. Mass Spectrum analysis for 15β-hydroxyboldenoen (**3a**). Protonated peak (**3c_1_**), ionized with sodium ion peak (**3c_2_**) and ionized with potassium ion peak (**3c_3_**) of 15β-hydroxyboldenoen.


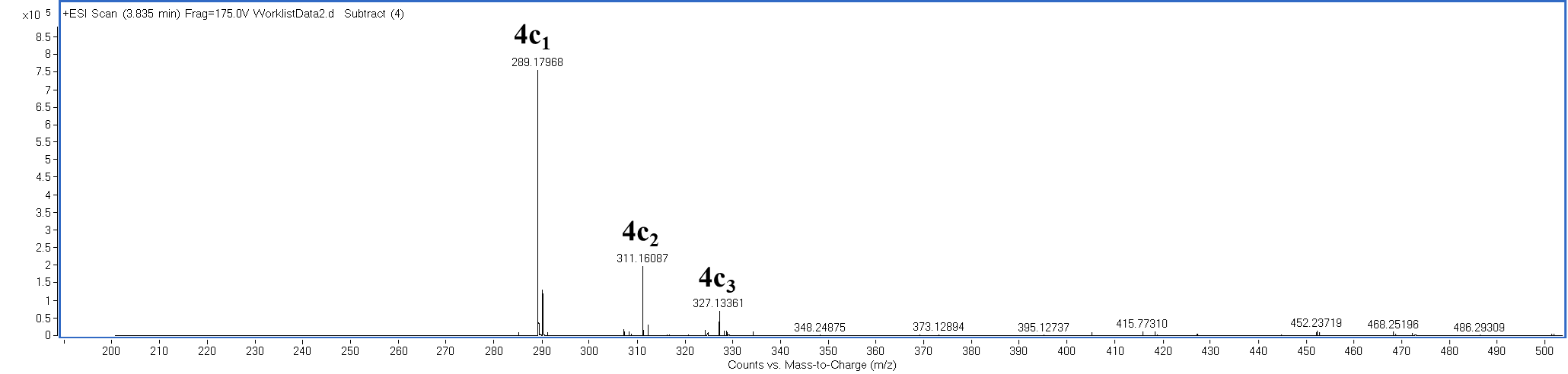


**Figure S17**. Mass Spectrum analysis for 15β-hydroxy-9(10)-dehydronandrolone (**4a**). Protonated peak (**4c_1_**), ionized with sodium ion peak (**4c_2_**) and ionized with potassium ion peak (**4c_3_**) of 15β-hydroxy 9(10)-dehydronandrolone.


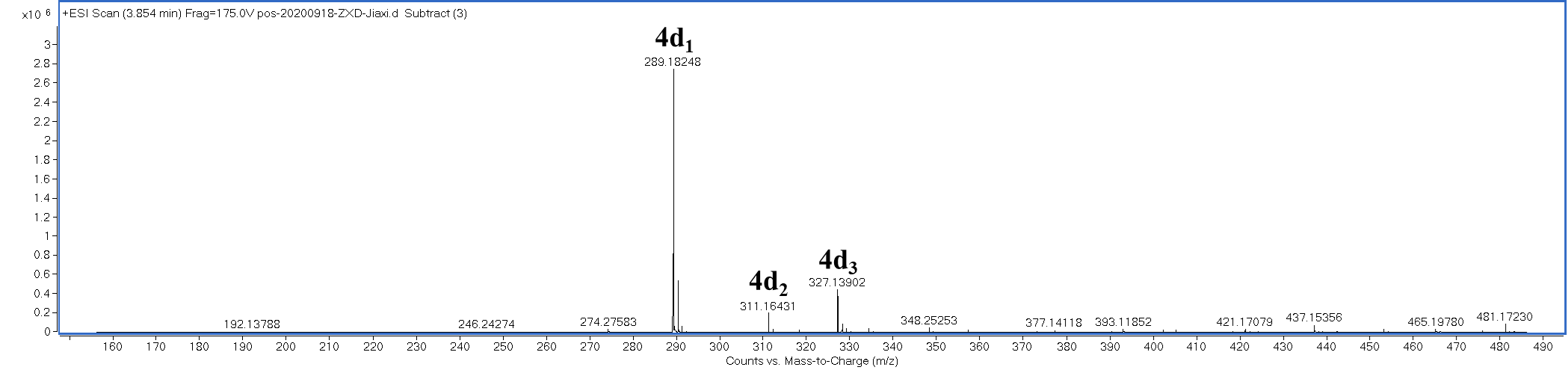


**Figure S18**. Mass Spectrum analysis for 16β-hydroxy-9(10)-dehydronandrolone (**4a**). Protonated peak (**4d_1_**), ionized with sodium ion peak (**4d_2_**) and ionized with potassium ion peak (**4d_3_**) of 16β-hydroxy 9(10)-dehydronandrolone..


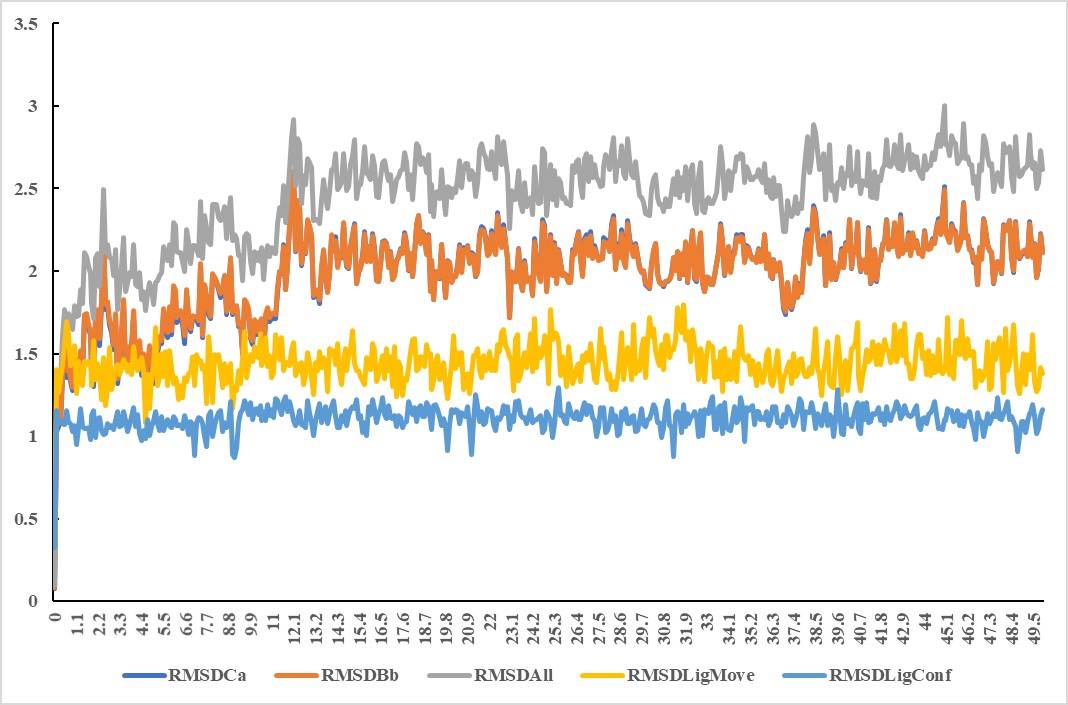


**Figure S19**. RMSD curve of the molecular dynamic (MD) simulation of the CYP109B1 crystal structure (PDB: 4RM4) for 50 ns.


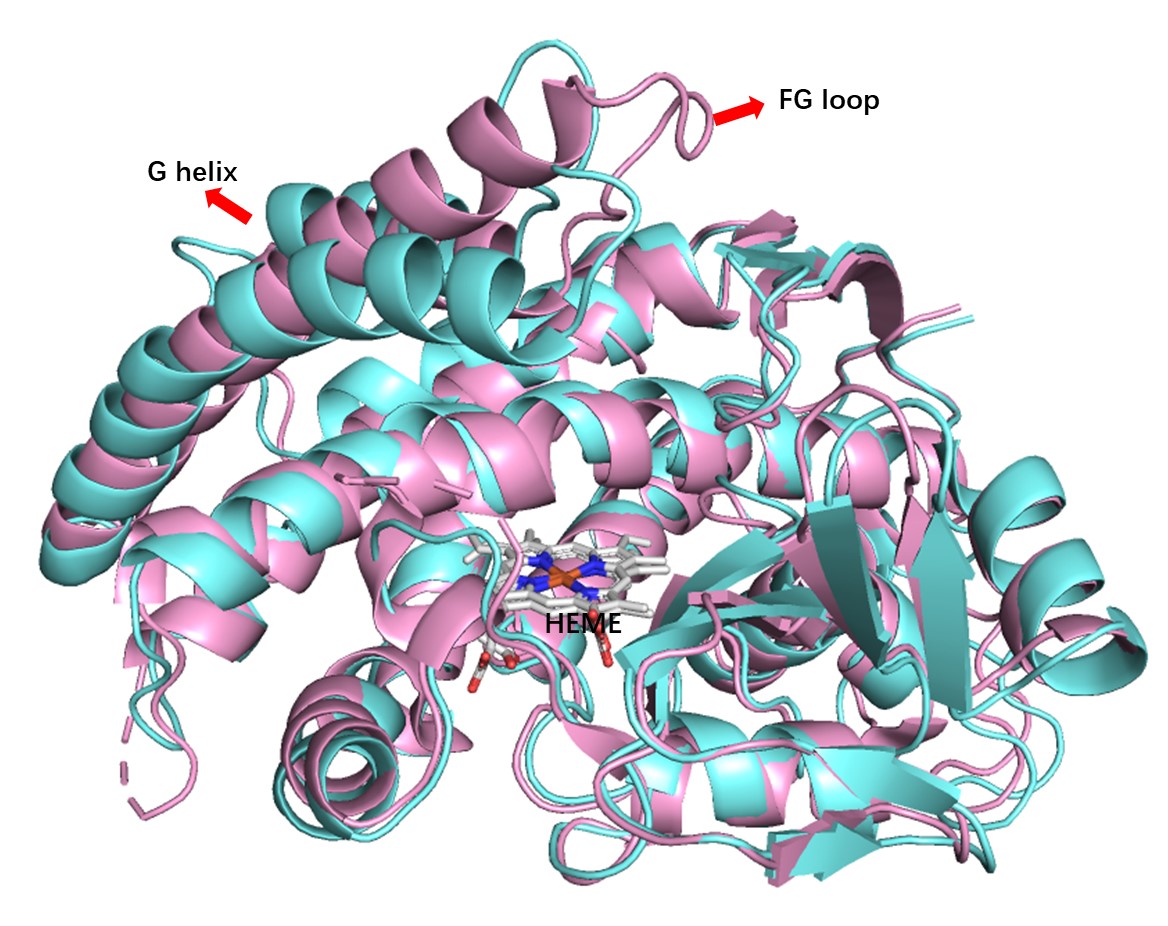


**Figure S20**. Superposition of substrate-free CYP109B1 before (pink) and after (cyan) molecular dynamic (MD) simulation. G helix, FG loop and HEME were labeled in black.


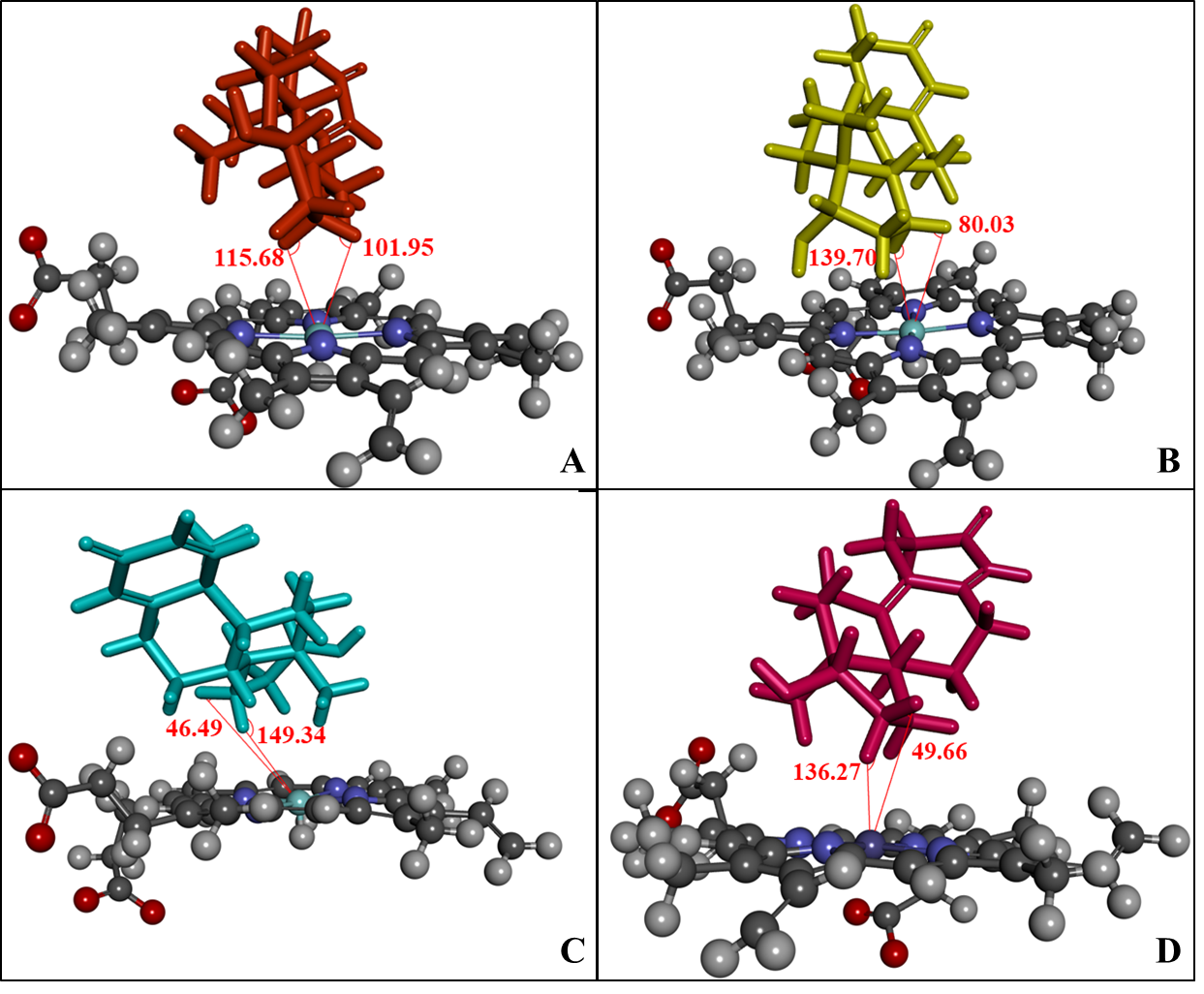


**Figure S21**. The dihedral angle between C-15 carbon atom, 15β hydrogen atom of steroid substrates and Fe atom of heme (Fe–H–C angle). **A**: The angle of the heme of CYP109B1 to testosterone (**1**). **B**: The angle of the heme of CYP109B1 to nandrolone (**2**). **C**: The dihedral angle of the heme of CYP109B1 to boldenone (**3**). **D**: The angle of the heme of CYP109B1 to 9(10) dehydronandrolone (**4**). The angle was lined together with value labeled in red


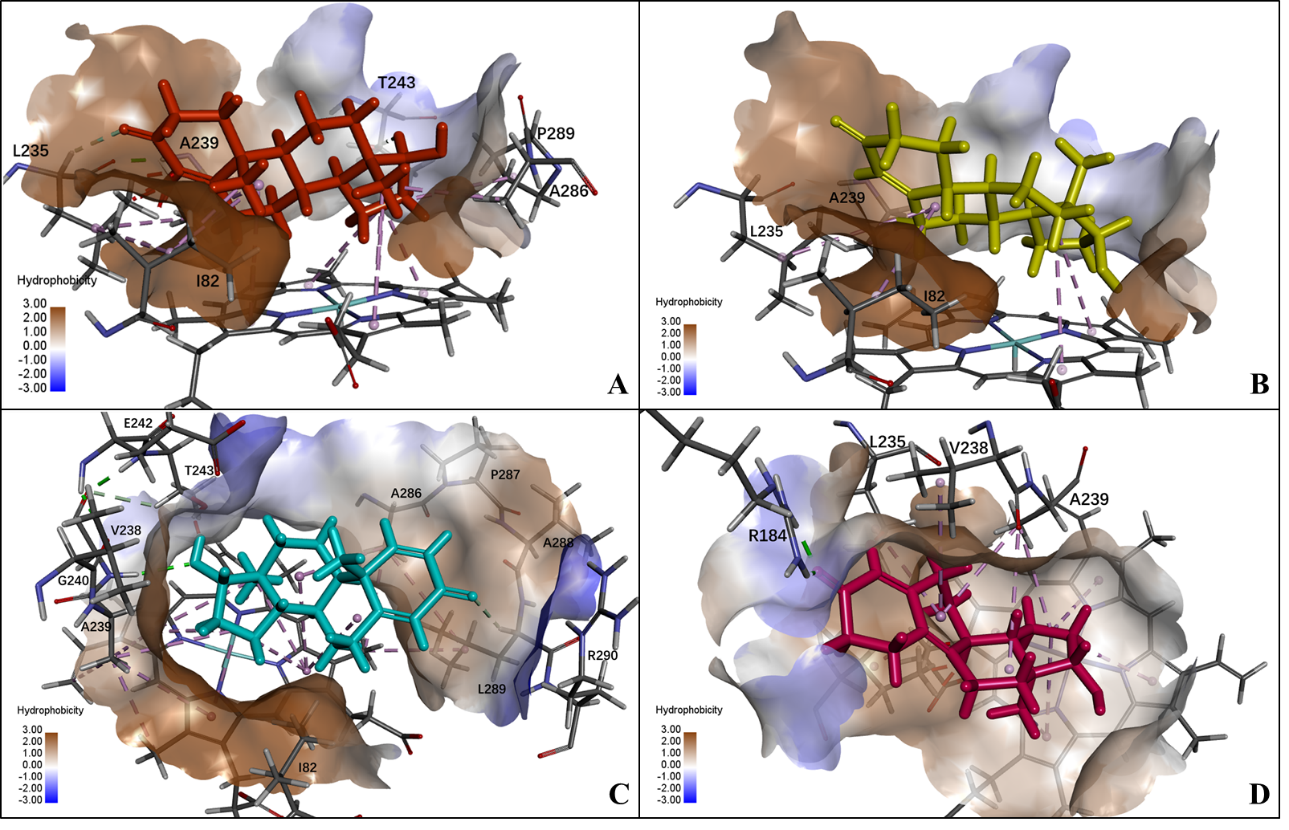


**Figure S22**. The hydrophobic interaction of steroid ligands with active sites in the pocket of CYP109B1. Active sites labeled in black, hydrophobic interaction between molecular with grey line. **A**: Hydrophobic interaction of CYP109B1 to testosterone (**1**). **B**: Hydrophobic interaction of CYP109B1 to nandrolone (**2**). **C**: Hydrophobic interaction of CYP109B1 to boldenone (**3**). **D**: Hydrophobic interaction of CYP109B1 to 9(10) dehydronandrolone (**4**).


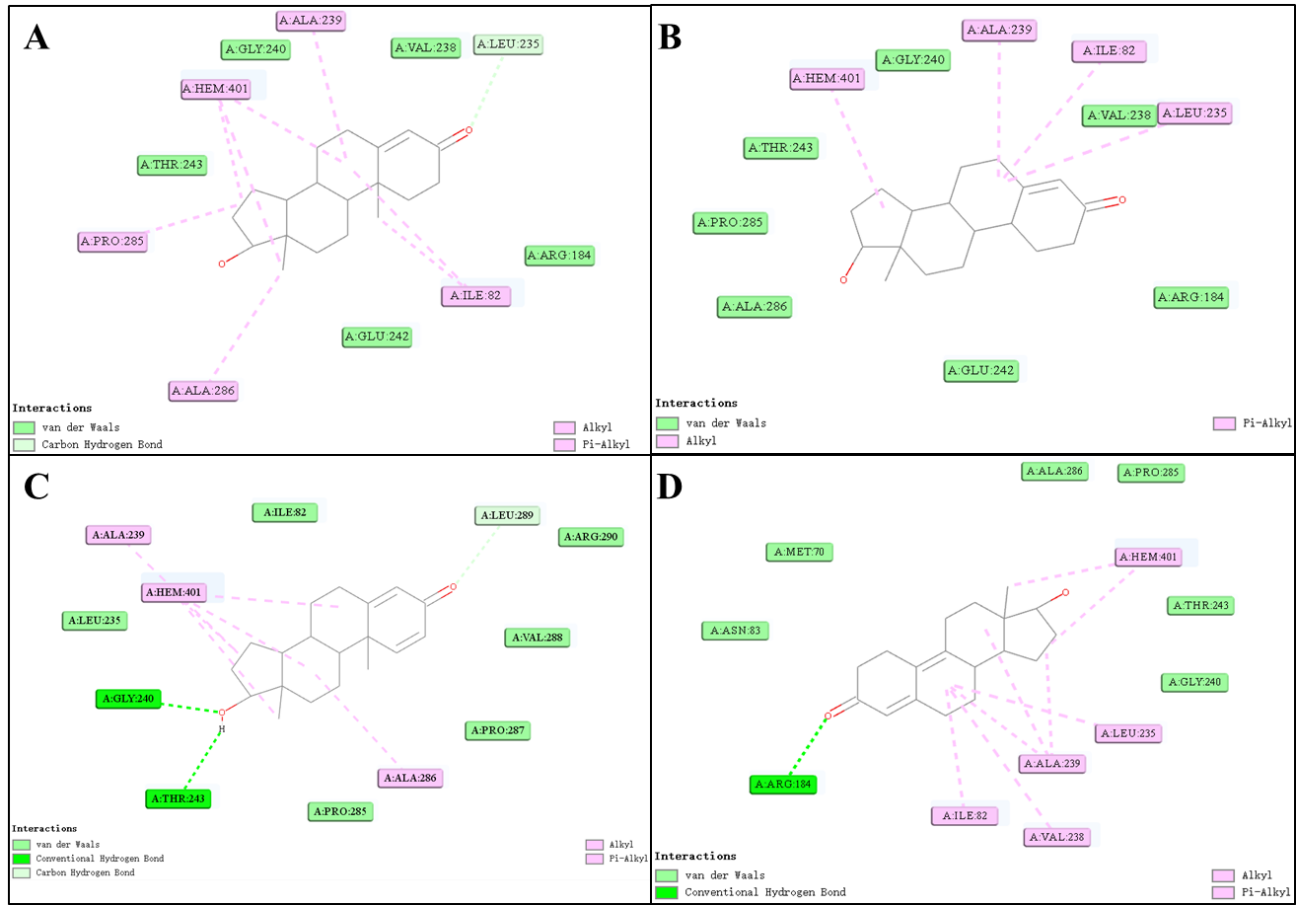


**Figure S23**. The planar graph of interactions between steroid ligands and CYP109B1. The residues in active sites were labeled in black with colors background based on interaction classification, and actual position of residues projected onto the plane. **A**: Interactions of CYP109B1 to testosterone (**1**). **B**: Interactions of CYP109B1 to nandrolone (**2**). **C**: Interactions of CYP109B1 to boldenone (**3**). **D**: Interactions of CYP109B1 to 9(10) dehydronandrolone (**4**).


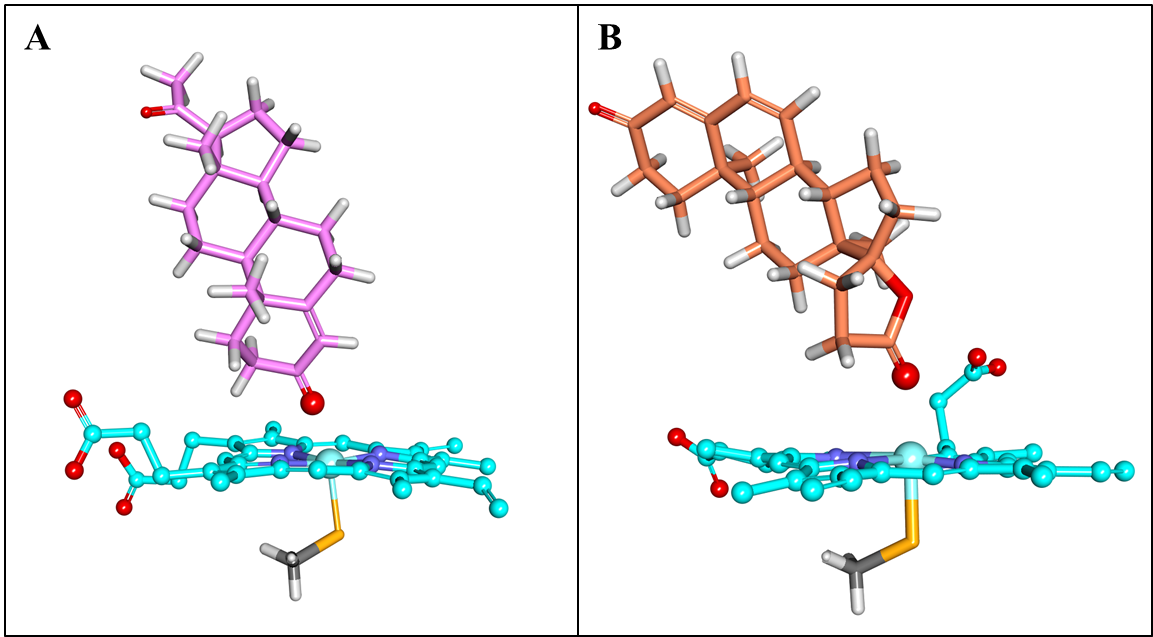


**Figure S24**. The representative conformations of CYP109B1-Progesterone (A) and CYP109B1-Canrenone (B) from the MD simulations. The HEM is colored in cyan, progesterone is colored in pink and canrenone is colored in orange.

**NMR spectra**

**Figure S25**

^1^H, 400MHz

**15β-hydroxy-testosterone**


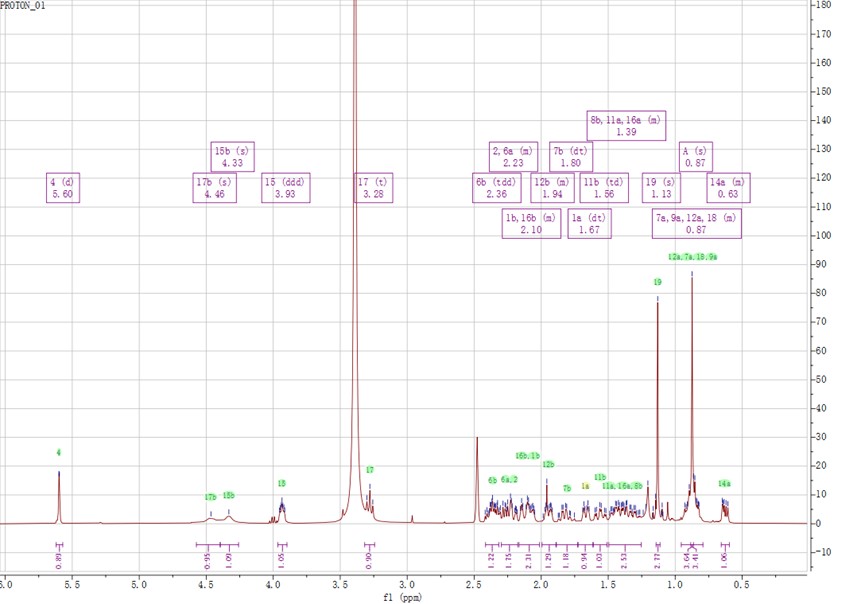


**Figure S26**

^13^C, 101MHz

**15β-hydroxy-testosterone**


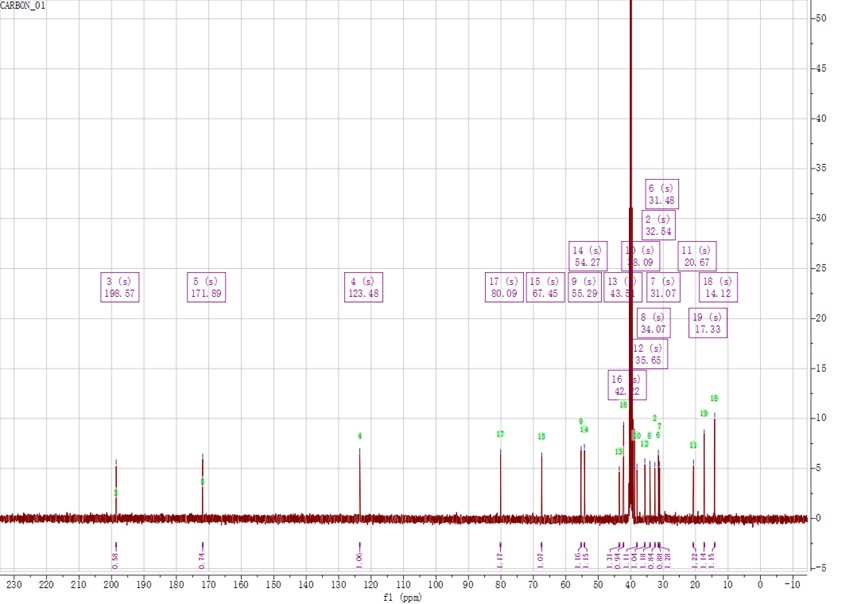


**Figure S27**

^1^H, 400MHz

**15β-hydroxy-nandrolone**

**
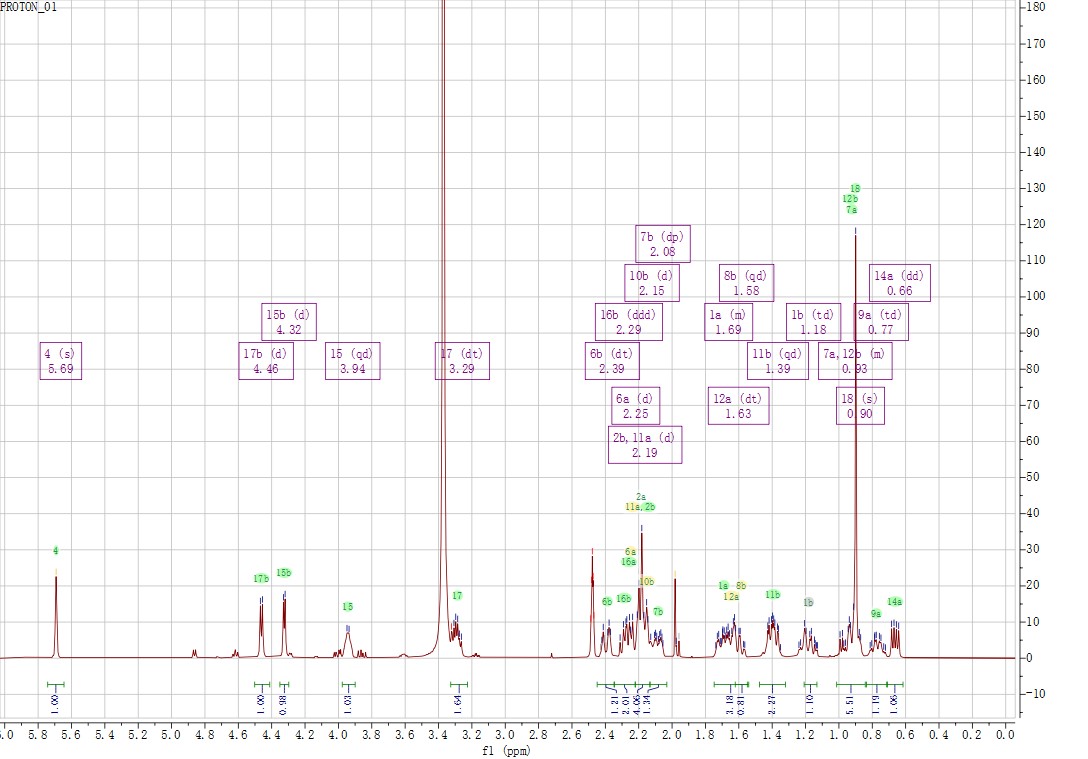
**

**Figure S28**

^13^C, 101MHz

**15β-hydroxy-nandrolone**

**
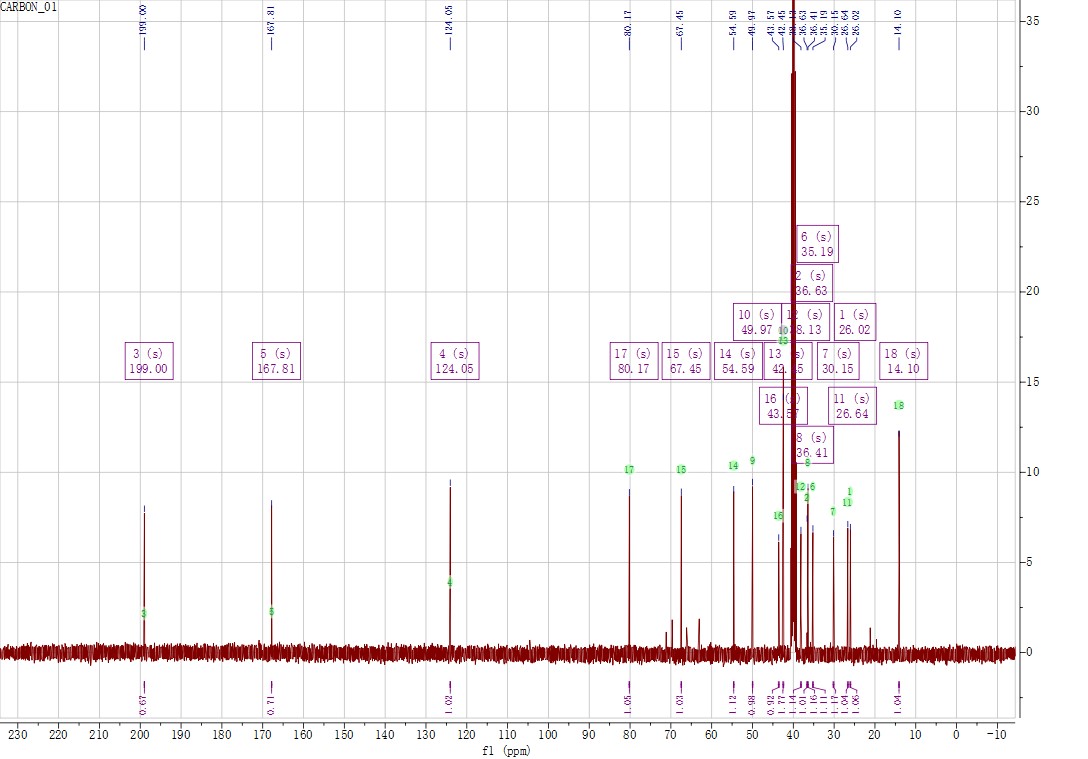
**

**Figure S29**

^1^H,^1^H,SHQC

**15β-hydroxy-nandrolone**

**
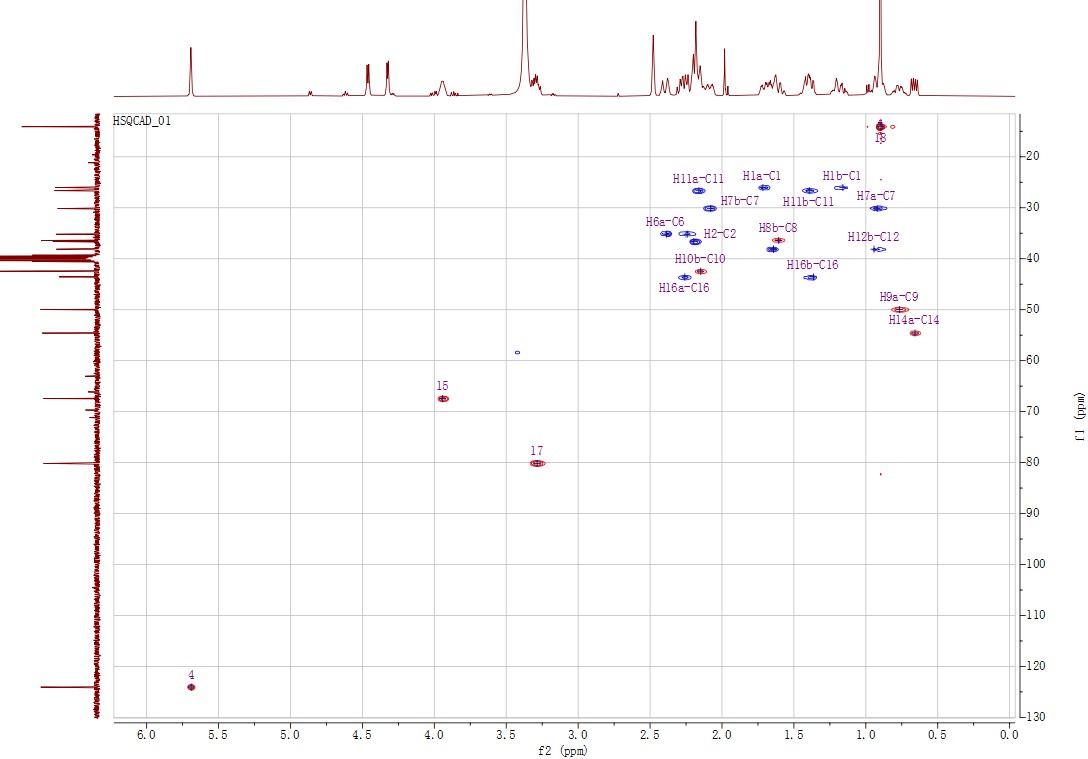
**

**Figure S30**

^1^H, 400MHz

**16β-hydroxy-nandrolone**


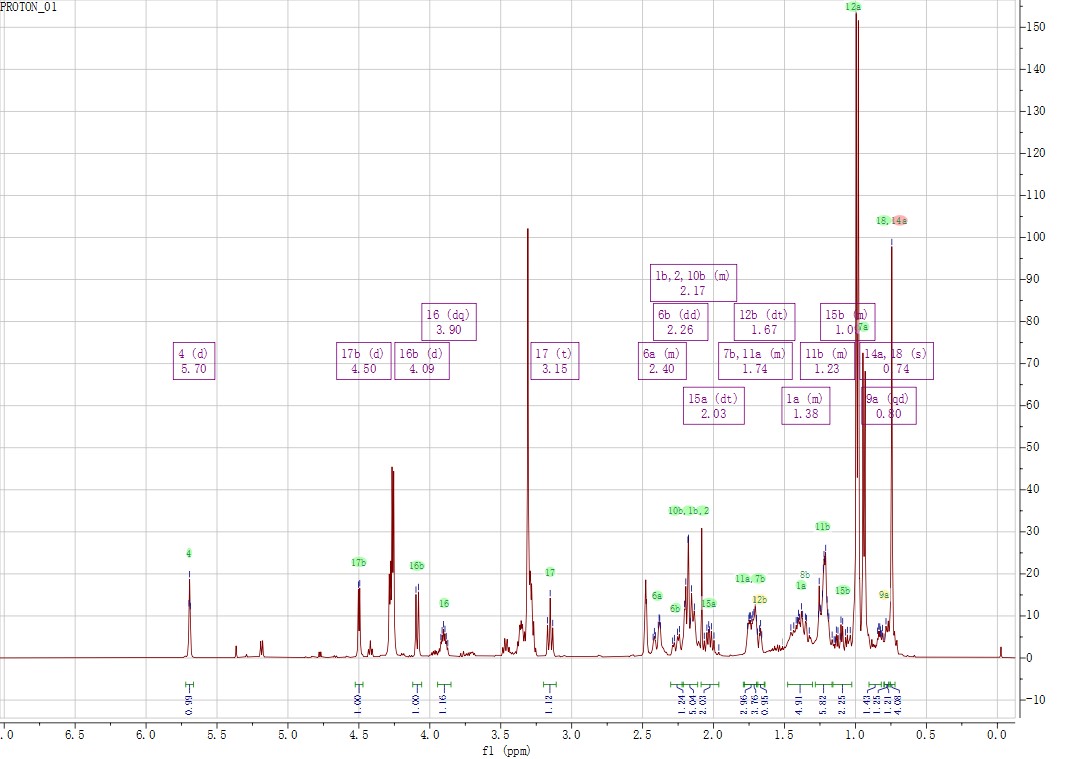


**Figure S31**

^14^C, 101MHz

**16β-hydroxy-nandrolone**


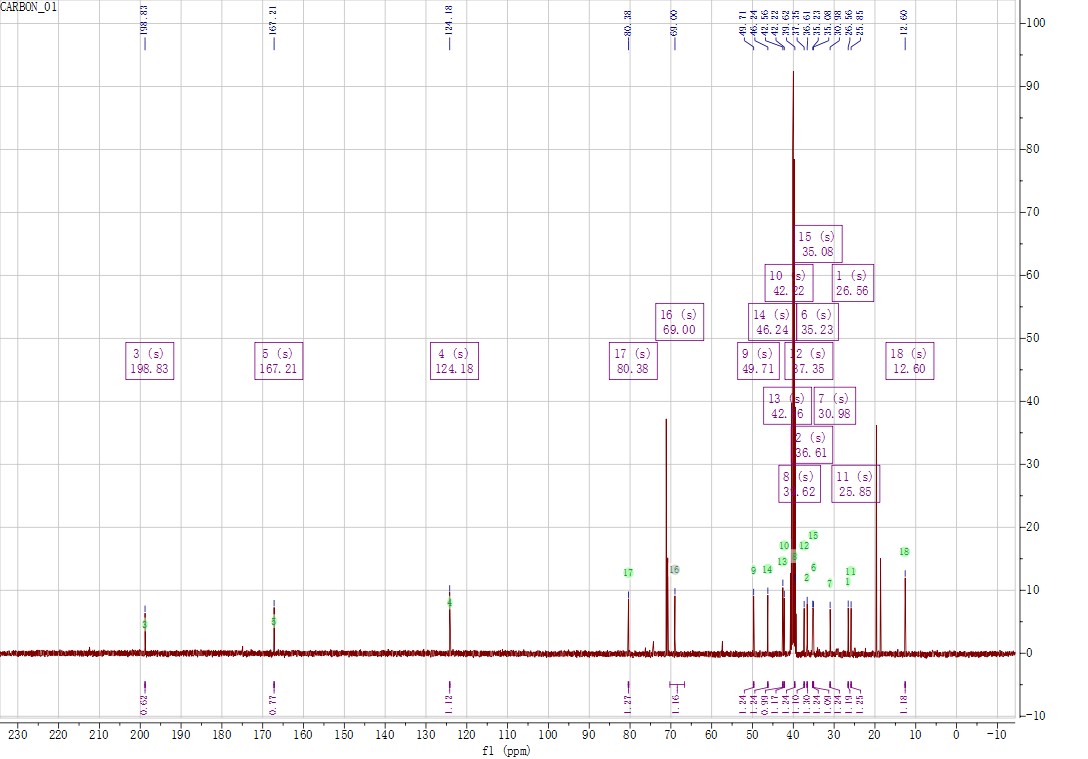


**Figure S32**

^1^H,^1^H,SHQC

**16β-hydroxy-nandrolone**


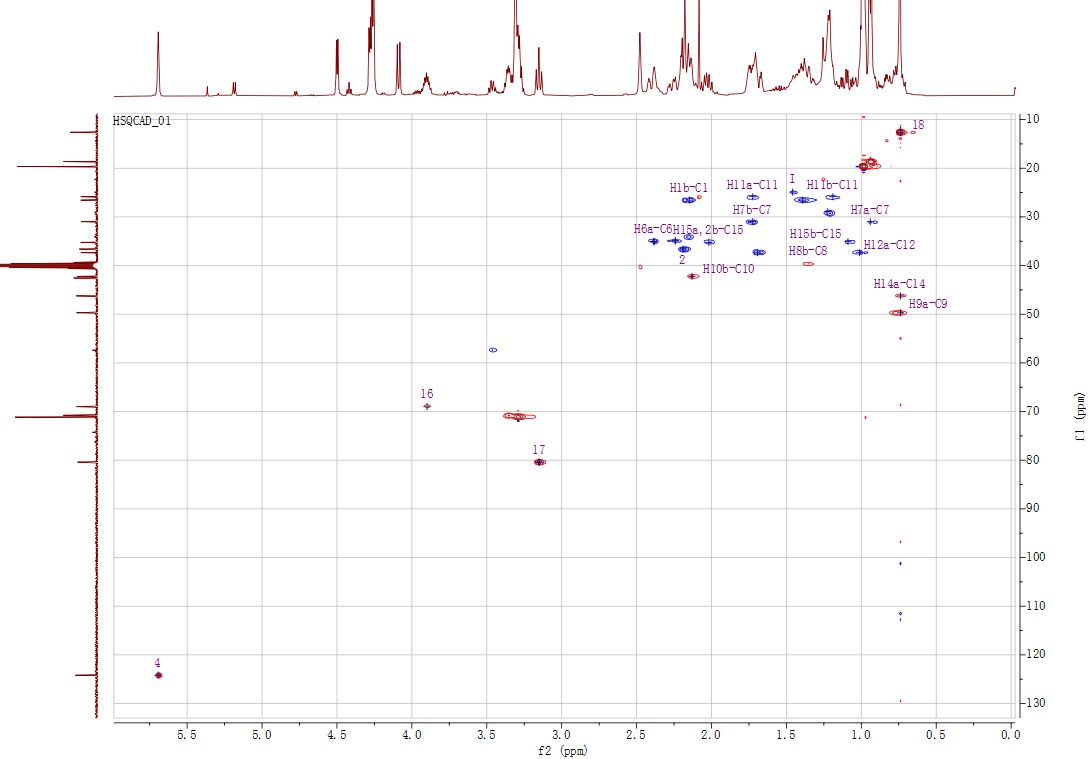


**Figure S33**

^1^H, 400MHz

**15β-hydroxy-boldenone**

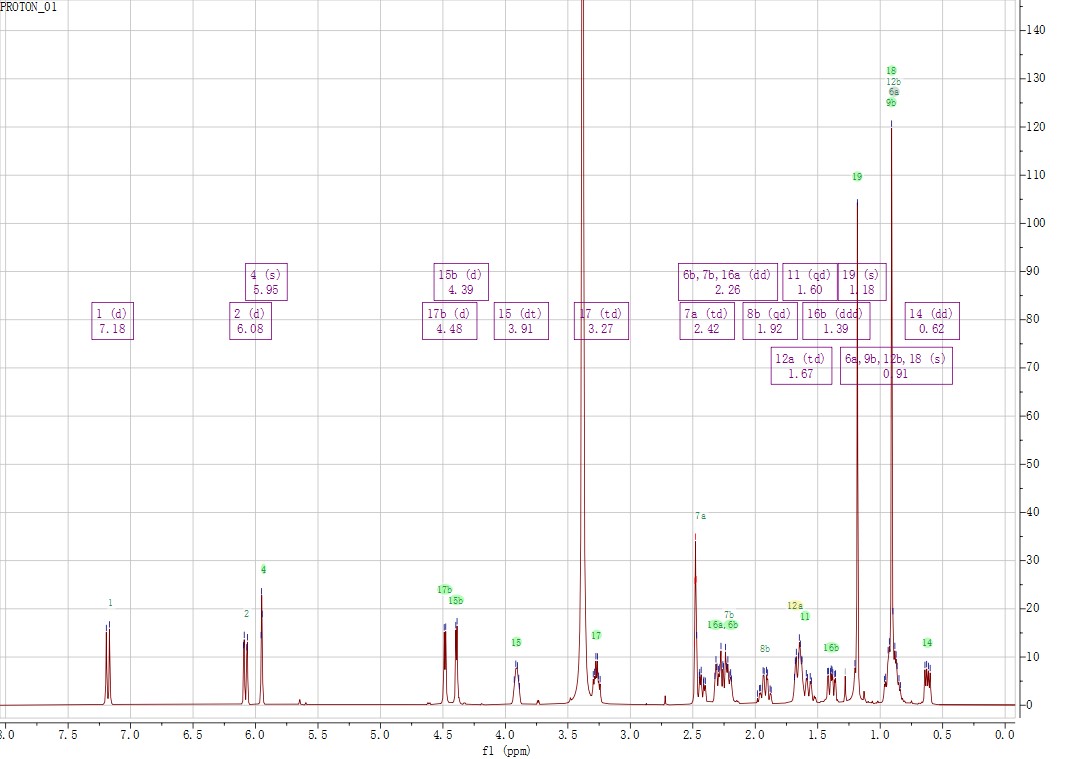


**Figure S34**

^13^C, 101MHz

**15β-hydroxy-boldenone**


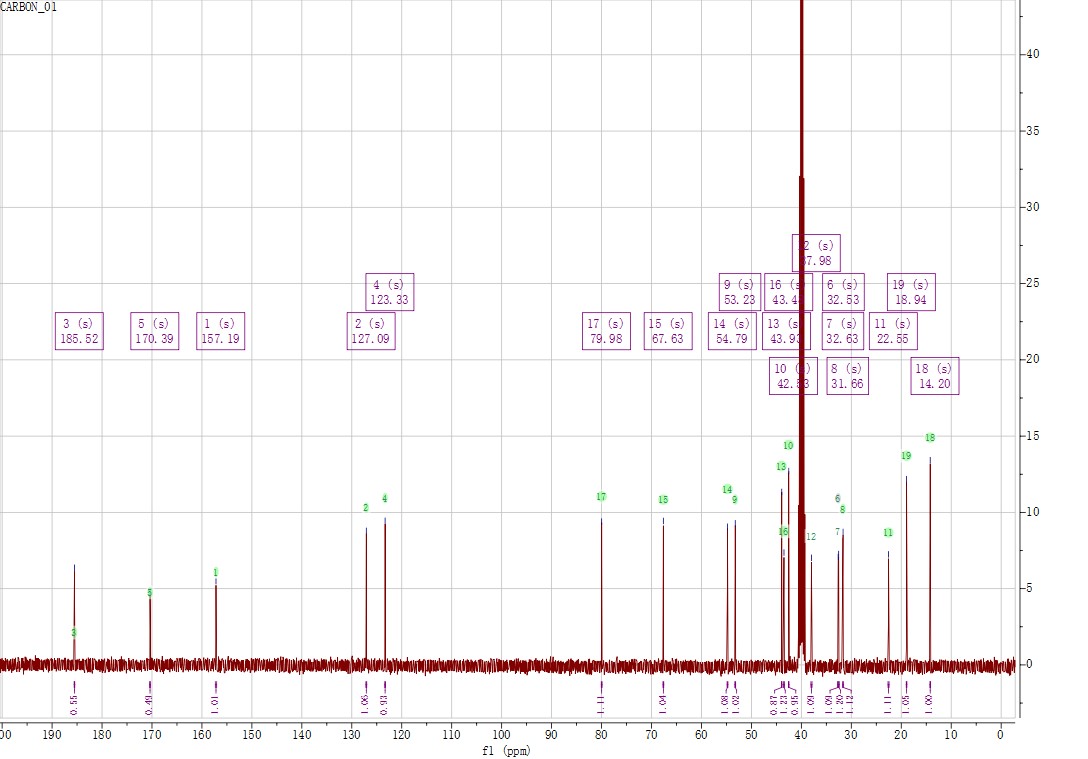


**Figure S35**

^1^H,^1^H,SHQC

**15β-hydroxy-boldenone**


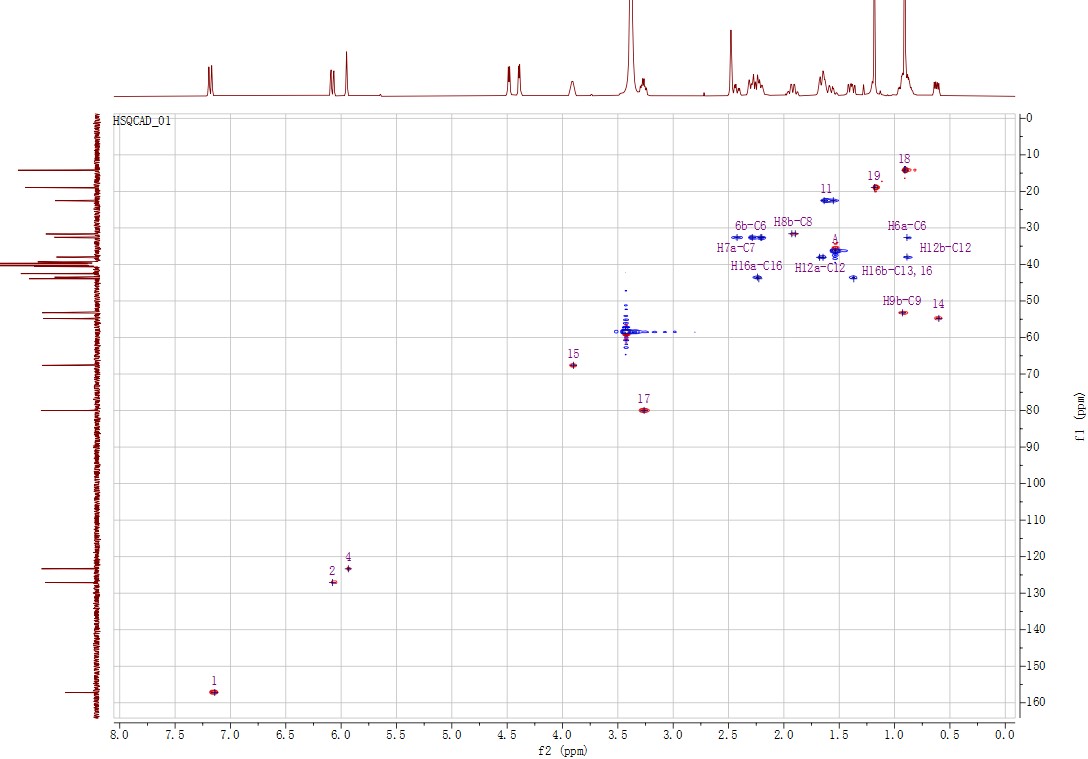


**Figure S36**

^1^H, 400MHz

**15β-hydroxy-9(10) dehydronandrolone**

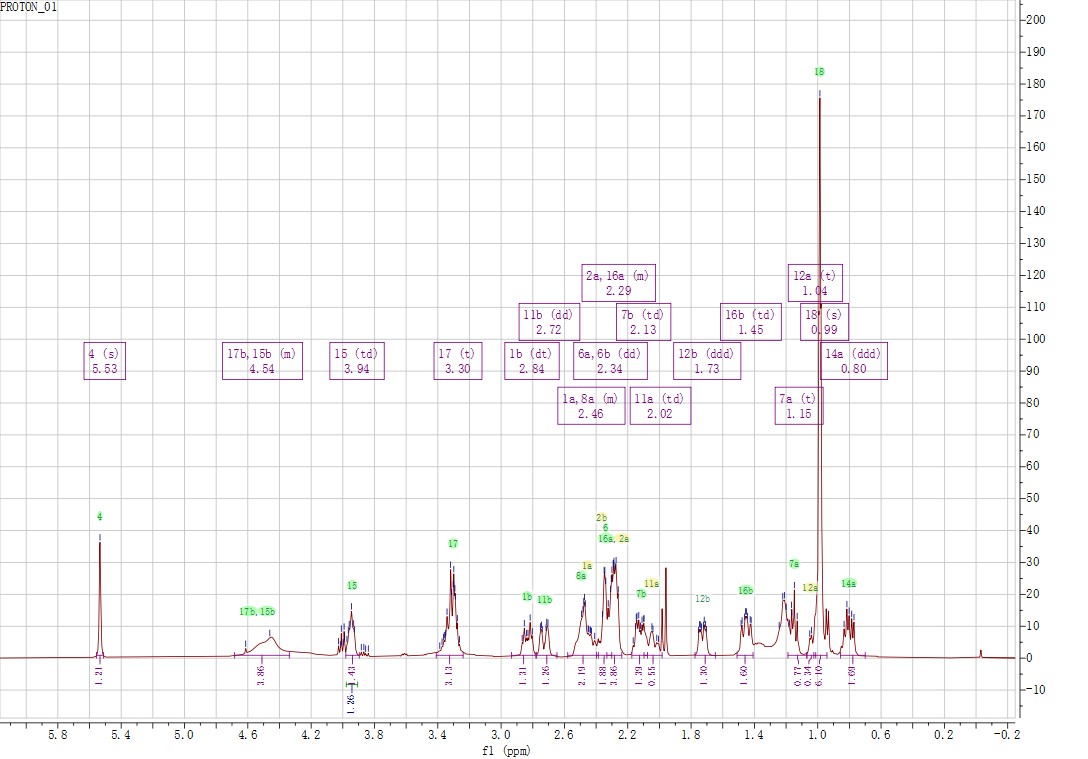


**Figure S37**

^13^C, 101MHz

**15β-hydroxy-9(10) dehydronandrolone**


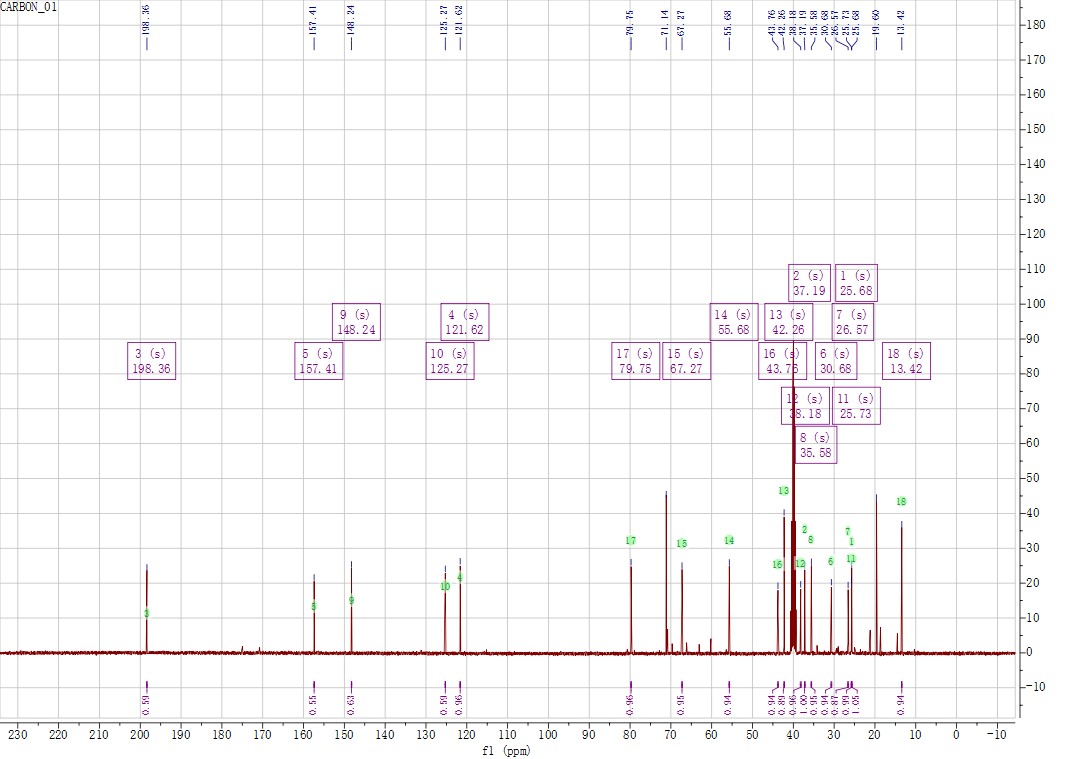


**Figure S38**

^1^H,^1^H,SHQC

**15β-hydroxy-9(10) dehydronandrolone**


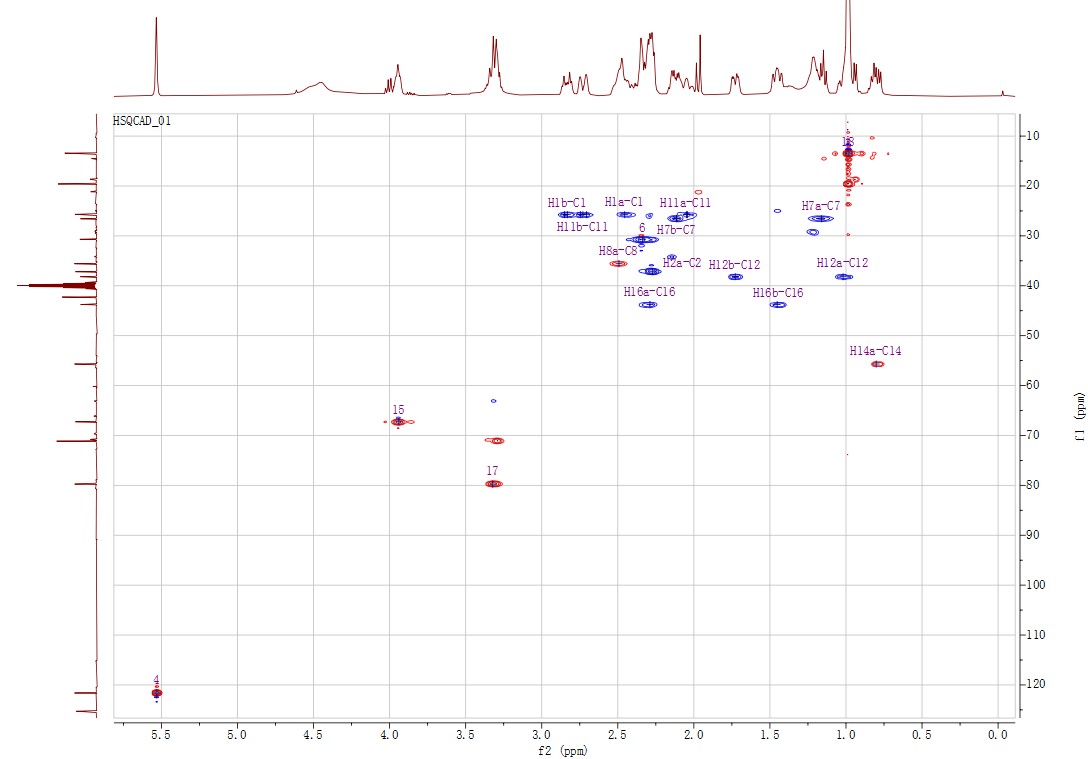


**Figure S39**

^1^H, 400MHz

**16β-hydroxy-9(10) dehydronandrolone**

**
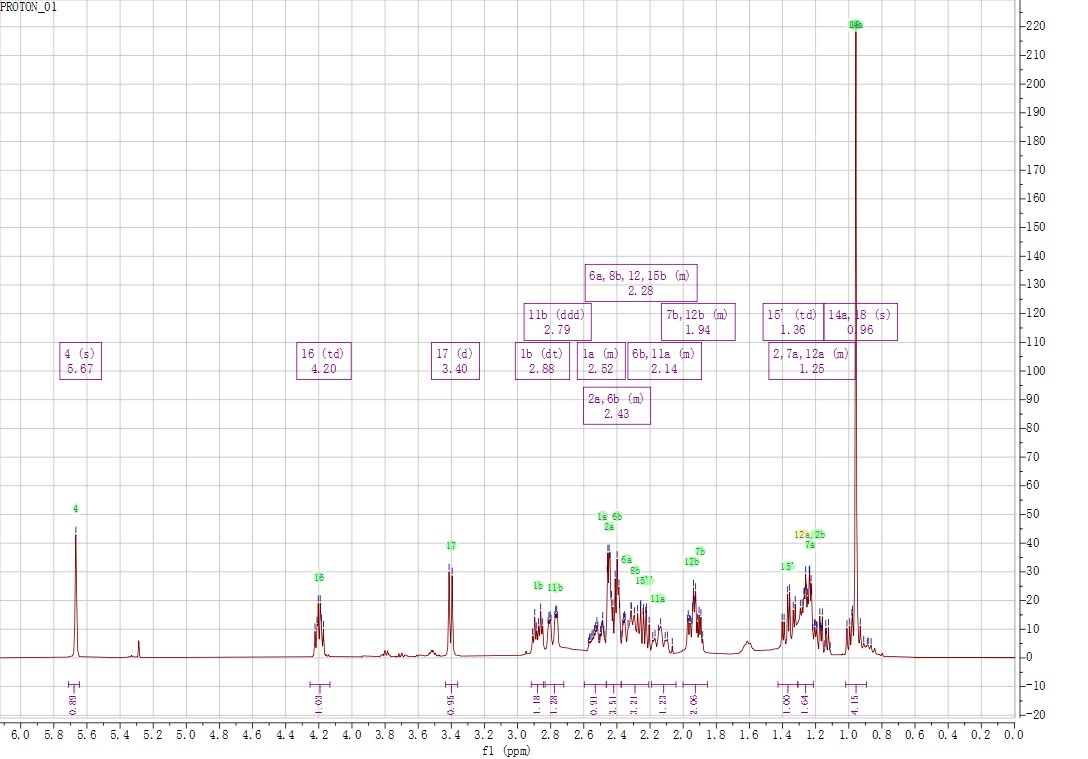
**

**Figure S40**

^13^C, 101MHz

**16β-hydroxy-9(10) dehydronandrolone**


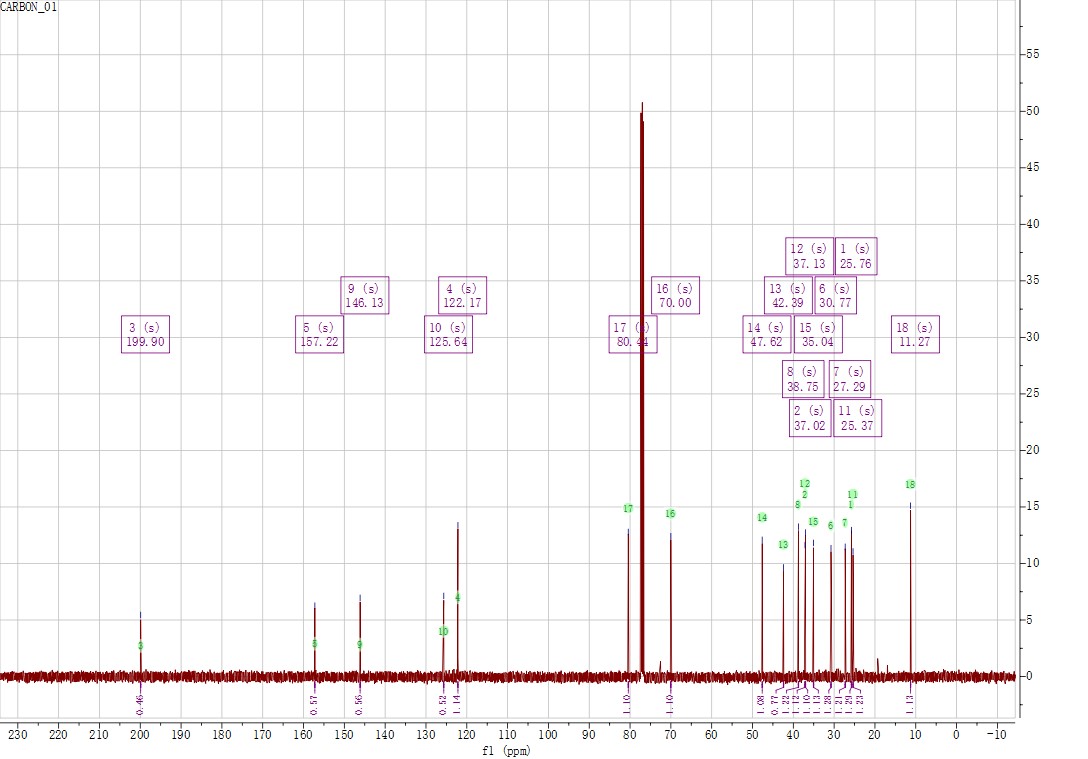


**Figure S41**

^1^H,^1^H,SHQC

**16β-hydroxy-9(10) dehydronandrolone**


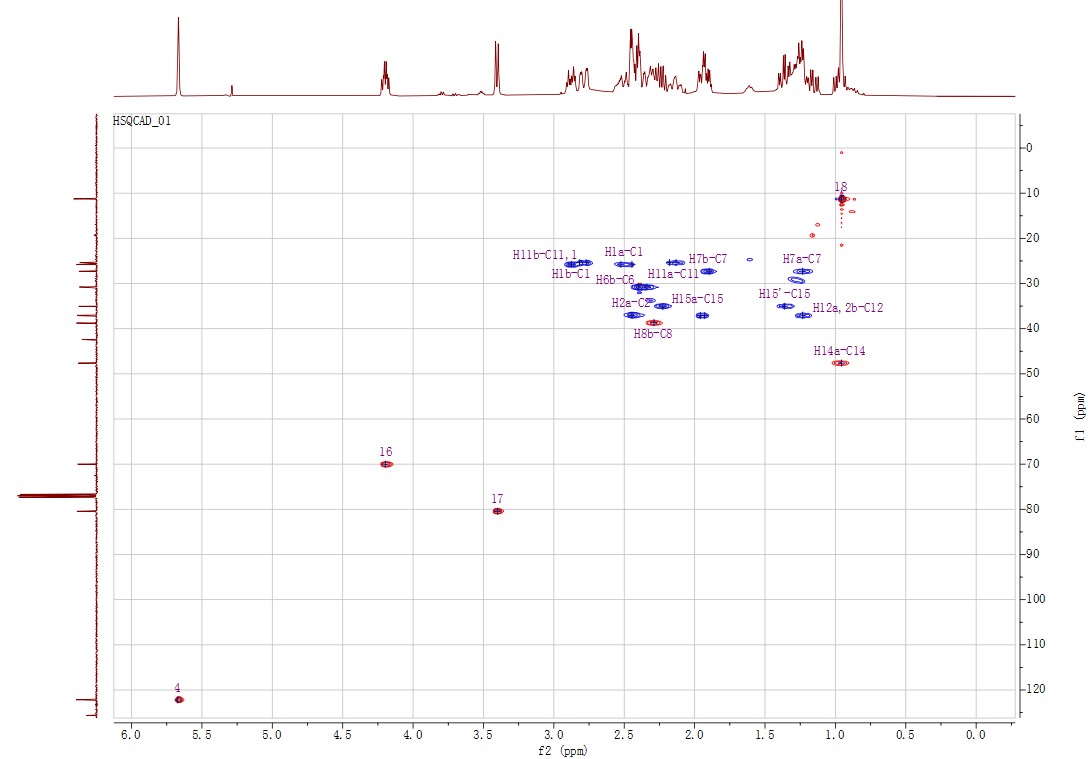


# Nucleotide sequence information:

**CYP109B1:**(*Bacillus subtilis* 168)

ATGAATGTGTTAAACCGCCGGCAAGCCTTGCAGCGAGCGCTGCTCAATGGGAAAAACAAACAGGATGCGTATCATCCGTTTCCATGGTATGAATCGATGAGAAAGGATGCGCCTGTTTCCTTTGATGAAGAAAACCAAGTGTGGAGCGTTTTTCTTTATGATGATGTCAAAAAAGTTGTTGGGGATAAAGAGTTGTTTTCCAGTTGCATGCCGCAGCAGACAAGCTCTATTGGAAATTCCATCATTAACATGGACCCGCCGAAGCATACAAAAATCCGTTCAGTCGTGAACAAAGCCTTTACTCCGCGCGTGATGAAGCAATGGGAACCGAGAATTCAAGAAATCACAGATGAACTGATTCAAAAATTTCAGGGGCGCAGTGAGTTTGACCTTGTTCACGATTTTTCATACCCGCTTCCGGTTATTGTGATATCTGAGCTGCTGGGAGTGCCTTCAGCGCATATGGAACAGTTTAAAGCATGGTCTGATCTTCTGGTCAGTACACCGAAGGATAAAAGTGAAGAAGCTGAAAAAGCCTTTTTGGAAGAACGAGATAAGTGTGAGGAAGAACTGGCCGCGTTTTTTGCCGGCATCATAGAAGAAAAGCGAAACAAACCGGAACAGGATATTATTTCTATTTTAGTGGAAGCGGAAGAAACAGGCGAGAAGCTGTCCGGTGAAGAGCTGATTCCGTTTTGCACGCTGCTGCTGGTGGCCGGAAATGAAACCACTACAAACCTGATTTCAAATGCGATGTACAGCATATTAGAAACGCCAGGCGTTTACGAGGAACTGCGCAGCCATCCTGAACTGATGCCTCAGGCAGTGGAGGAAGCCTTGCGTTTCAGAGCGCCGGCCCCGGTTTTGAGGCGCATTGCCAAGCGGGATACGGAGATCGGGGGGCACCTGATTAAAGAAGGTGATATGGTTTTGGCGTTTGTGGCATCGGCAAATCGTGATGAAGCAAAGTTTGACAGACCGCACATGTTTGATATCCGCCGCCATCCCAATCCGCATATTGCGTTTGGCCACGGCATCCATTTTTGCCTTGGGGCCCCGCTTGCCCGTCTTGAAGCAAATATCGCGTTAACGTCTTTGATTTCTGCTTTTCCTCATATGGAGTGCGTCAGTATCACTCCGATTGAAAACAGTGTGATATACGGATTAAAGAGCTTCCGTGTGAAAATGTGA

**Fdr_0978:** (*Synechococcus elongates* PCC7942)

ATGTTGAATGCGAGTGTGGCTGGCGGAGCAGCTACCACCACCTATGGCAACCGGCTCTTTATCTATGAAGTGATCGGTCTGCGCCAAGCCGAGGGCGAACCGTCCGACAGCTCAATCCGCCGTAGTGGCAGCACCTTCTTCAAGGTGCCTTACAGCCGGATGAATCAAGAAATGCAACGGATTTTGCGCCTTGGCGGCAAAATCGTTAGCATCCGGCCTGCGGAGGAAGCAGCCGCGAATAATGGTGCGGCTCCTCTACAGGCAGCAGCTGAAGAACCTGCTGCAGCACCAACCCCCGCTCCGGCTGCCAAAAAACATTCAGCCGAAGACGTGCCTGTCAATATCTACCGGCCTAACAAGCCTTTCGTAGGCAAGGTGCTCTCGAACGAGCCCTTGGTTCAAGAAGGCGGGATTGGTGTTGTGCAGCACCTCACCTTCGATATTTCGGAAGGCGATCTGCGCTACATCGAAGGTCAAAGTATCGGGATTATCCCGGATGGCACCGATGACAAAGGCAAGCCGCACAAGCTCCGTCTTTACTCGATCGCATCCACTCGCCACGGCGACCACGTGGATGACAAAACCGTCTCGCTGTGCGTGCGCCAGCTGCAGTACCAGAACGAAGCCGGCGAAACGATTAATGGCGTCTGCTCGACTTTCCTCTGTGGTCTGAAGCCAGGCGATGACGTCAAGATCACCGGTCCTGTGGGCAAAGAAATGCTCCTACCGGCGGACACAGACGCCAACGTGATCATGATGGGTACTGGCACCGGGATTGCTCCGTTCCGAGCCTACCTATGGCGGATGTTTAAAGACAACGAGCGAGCCATCAACAGCGAGTATCAATTCAACGGCAAGGCTTGGTTGATCTTCGGGATTCCGACGACCGCCAACATCCTCTACAAAGAGGAGCTGGAAGCGCTGCAGGCTCAGTATCCAGATAACTTCCGCCTGACCTACGCGATCAGCCGCGAGCAGAAAAATGAAGCGGGCGGCCGGATGTACATCCAAGACCGCGTCGCTGAACATGCTGACGAGATCTGGAACCTACTCAAGGACGAAAAAACCCACGTCTATATCTGTGGTTTGCGTGGCATGGAAGATGGGATCGATCAAGCCATGACCGTCGCAGCTGCCAAGGAAGATGTGGTTTGGTCTGACTACCAACGCACCCTCAAGAAAGCGGGTCGTTGGCATGTTGAAACCTACTAG

**Fdx_1499:** (*Synechococcus elongates* PCC7942)

ATGGCAACCTACAAGGTTACGCTCGTCAATGCTGCCGAAGGCTTGAACACCACGATCGACGTGGCTGACGATACCTACATCTTGGACGCCGCTGAAGAGCAAGGCATTGACCTGCCTTACTCCTGCCGTGCTGGTGCTTGCTCGACCTGTGCTGGCAAAGTCGTCTCTGGTACCGTCGACCAATCGGATCAATCCTTCTTGGATGACGACCAAATTGCAGCAGGCTTTGTCCTGACCTGCGTCGCCTATCCGACCTCCGATGTGACGATCGAAACCCACAAAGAAGAAGACCTCTACTAA

**FNR:** (*spinach*, E.C.1.18.1.2)

ATGCAGATCGCCTCTGATGTGGAGGCACCTcCACCTGCTCCTGCTAAGGTAGAGAAACATTCAAAGAAAATGGAGGAAGGCATTACAGTTAACAAGTTTAAGCCTAAGAcCCCTTACGTTGGAAGATGTCTTCTTAACACCAAAATTACTGGGGATGATGCACCCGGAGAGACCTGGCACATGGTTTTTTCCCATGAAGGAGAGATCCCTTACAGAGAAGGGCAATCCGTTGGGGTTATTCCAGATGGGGAAGACAAGAATGGAAAGCCCCATAAGTTGAGATTGTACTCGATCGCCAGCAGTGCTCTTGGTGATTTTGGTGATGCTAAATCTGTTTCGTTGTGTGTAAAACGACTCATCTACACCAATGACGCTGGAGAGACGATCAAGGGAGTCTGCTCCAACTTCTTGTGTGACTTGAAACCCGGTGCTGAAGTGAAGTTAACAGGACCAGTTGGAAAGGAGATGCTCATGCCCAAAGACCCTAACGCGACAATTATCATGCTTGGAACTGGAACTGGGATTGCTCCTTTCCGTTCATTCTTGTGGAAGATGTTCTTCGAAAAGCATGATGATTACAAGTTTAACGGCTTGGCTTGGCTTTTCTTGGGTGTACCCACAAGCAGTTCTCTTCTCTACAAAGAGGAATTTGAGAAGATGAAGGAAAAGGCTCCAGACAACTTCAGGCTGGATTTTGCAGTGAGCAGAGAGCAAACTAACGAGAAAGGGGAGAAGATGTACATTCAAACCCGAATGGCACAATACGCAGTTGAGCTATGGGAAATGTTGAAGAAAGATAATACTTATTTCTACATGTGTGGTCTCAAGGgAATGGAAAAGGGAATTGACGACATTATGGtTTCATTGGCTGCTGCAGAAGGCATTGATTGGATTGAATACAAGAGGCAGTTGAAGAAGGCAGAACAATGGAACGTTGAAGTCTACTAA

**Fd I:** (*spinach*, E.C.1.18.1.2)

ATGGCCGCCTACAAGGTGACCCTGGTGACCCCGACCGGCAACGTGGAGTTCCAGTGCCCGGACGACGTGTACATCCTGGACGCCGCAGAAGAAGAGGGGATCGACCTGCCGTACAGCTGTCGCGCAGGCAGTTGCAGCAGCTGCGCAGGTAAGCTGAAAACCGGCAGCCTGAACCAGGACGACCAGAGCTTCCTGGACGACGACCAGATCGACGAGGGCTGGGTTCTGACATGCGCCGCCTACCCGGTGAGCGATGTGACCATCGAGACCCACAAAGAAGAGGAGCTGACCGCCTAA

**Fpr:** (*E. coli*)

ATGGCTGATTGGGTAACAGGCAAAGTCACTAAAGTGCAGAACTGGACCGACGCCCTGTTTAGTCTCACCGTTCACGCCCCCGTGCTTCCGTTTACCGCCGGGCAATTTACCAAGCTTGGCCTTGAAATCGACGGCGAACGCGTCCAGCGCGCCTACTCCTATGTAAACTCGCCCGATAATCCCGATCTGGAGTTTTACCTGGTCACCGTCCCCGATGGCAAATTAAGCCCACGACTGGCGGCACTGAAACCAGGCGATGAAGTGCAGGTGGTTAGCGAAGCGGCAGGATTCTTTGTGCTCGATGAAGTGCCGCACTGCGAAACGCTATGGATGCTGGCAACCGGTACAGCGATTGGCCCTTATTTATCGATTCTGCAACTAGGTAAAGATTTAGATCGCTTCAAAAATCTGGTCCTGGTGCACGCCGCACGTTATGCCGCCGACTTAAGCTATTTGCCACTGATGCAGGAACTGGAAAAACGCTACGAAGGAAAACTGCGCATTCAGACGGTGGTCAGTCGGGAAACGGCAGCGGGGTCGCTCACCGGACGGATACCGGCATTAATTGAAAGTGGGGAACTGGAAAGCACGATTGGCCTGCCGATGAATAAAGAAACCAGCCATGTGATGCTGTGCGGCAATCCACAGATGGTGCGCGATACACAACAGTTGCTGAAAGAGACCCGGCAGATGACGAAACATTTACGTCGCCGACCGGGCCATATGACAGCGGAGCATTACTGGTAA

**YkuN**: (*B. subtilis*)

ATGGCTAAAGCCTTGATTACATATGCCAGCATGTCAGGAAATACAGAAGACATTGCCTTCATAATAAAAGATACGCTTCAGGAATATGAGTTGGATATCGATTGTGTCGAGATAAATGATATGGATGCGTCTTGTTTAACCTCCTATGATTATGTACTGATTGGCACCTATACATGGGGGGACGGCGATTTGCCCTACGAAGCGGAGGATTTTTTCGAAGAGGTCAAACAGATTCAGCTTAATGGTTTAAAAACAGCCTGCTTCGGGTCTGGCGATTATTCTTATCCAAAGTTTTGCGAAGCGGTGAATTTGTTCAATGTCATGCTGCAAGAGGCGGGAGCTGCTGTTTACCAGGAAACACTAAAAATTGAATTAGCGCCTGAAACAGATGAAGATGTGGAAAGCTGCCGAGCGTTTGCGAGAGGTTTTCTTGCATGGGCAGATTATATGAACAAGGAAAAAATCCATGTTTCATAA

**YkuP**: (*B. subtilis*)

ATGGCGAAGATTTTGCTCGTTTATGCAACAATGTCAGGCAACACTGAAGCTATGGCAGATTTGATTGAAAAGGGGCTTCAGGAGGCGTTAGCAGAAGTAGACCGTTTCGAAGCAATGGATATTGATGATGCCCAGCTGTTTACCGATTATGACCATGTCATAATGGGAACCTACACGTGGGGAGACGGAGATCTGCCTGATGAATTTTTAGATCTTGTTGAAGACATGGAGGAGATTGATTTTTCCGGCAAAACATGCGCTGTATTCGGTTCCGGTGATACAGCATATGAATTTTTCTGCGGAGCGGTTGATACGCTAGAGGCAAAAATAAAAGAACGCGGTGGAGACATTGTGCTGCCTTCGGTAAAAATCGAAAATAATCCAGAAGGTGAAGAAGAGGAAGAATTAATAAACTTCGGGAGACAATTCGCAAAGAAAAGCGGGTGCGCTGTCTGA

**RhF reductase region:**(*Rhodococcus sp*. Strain NCIMB 9784)

GTGCTGCACCGGCATCAACCGGTCACCATCGGAGAACCCGCCGCCCGGGCGGTGTCCCGCACCGTCACCGTCGAGCGCCTGGACCGGATCGCCGACGACGTGCTGCGCCTCGTCCTGCGCGACGCCGGCGGAAAGACATTACCCACGTGGACTCCCGGCGCCCATATCGACCTCGACCTCGGCGCGCTGTCGCGCCAGTACTCCCTGTGCGGCGCGCCCGATGCGCCGAGCTACGAGATTGCCGTGCACCTGGATCCCGAGAGCCGCGGCGGTTCGCGCTACATCCACGAACAGCTCGAGGTGGGAAGCCCGCTCCGGATGCGCGGCCCTCGGAACCATTTCGCGCTCGACCCCGGCGCCGAGCACTACGTGTTCGTCGCCGGCGGCATCGGCATCACCCCAGTCCTGGCCATGGCCGACCACGCCCGCGCCCGGGGGTGGAGCTACGAACTGCACTACTGCGGCCGAAACCGTTCCGGCATGGCCTATCTCGAGCGTGTCGCCGGGCACGGTGACCGGGCCGCCCTGCACGTGTCCGAGGAAGGCACCCGGATCGACCTCGCCGCCCTCCTCGCCGAGCCCGCCCCCGGCGTCCAGATCTACGCGTGCGGGCCCGGGCGGCTGCTCGCCGGACTCGAGGACGCGAGCCGGAACTGGCCCGACGGGGCGCTGCACGTCGAGCACTTCACCTCGTCCCTCGCGGCGCTCGATCCGGACGTCGAGCACGCCTTCGACCTCGAACTGCGTGACTCGGGGCTGACCGTGCGGGTCGAACCCACCCAGACCGTCCTCGACGCGTTGCGCGCCAACAACATCGACGTGCCCAGCGACTGCGAGGAAGGCCTCTGCGGCTCGTGCGAGGTCGCCGTCCTCGACGGCGAGGTCGACCATCGCGACACGGTGCTGACCAAGGCCGAGCGGGCGGCGAACCGGCAGATGATGACCTGCTGCTCGCGTGCCTGTGGCGACCGGCTGGCCCTGCGACTCTGA

**BM3 reductase region:**(*B.megatherium*)

CCTTCACCTAGCACTGAACAGTCTGCTAAAAAAGTACGCAAAAAGGCAGAAAACGCTCATAATACGCCGCTGCTTGTGCTATACGGTTCAAATATGGGAACAGCTGAAGGAACGGCGCGTGATTTAGCAGATATTGCAATGAGCAAAGGATTTGCACCGCAGGTCGCAACGCTTGATTCACACGCCGGAAATCTTCCGCGCGAAGGAGCTGTATTAATTGTAACGGCGTCTTATAACGGTCATCCGCCTGATAACGCAAAGCAATTTGTCGACTGGTTAGACCAAGCGTCTGCTGATGAAGTAAAAGGCGTTCGCTACTCCGTATTTGGATGCGGCGATAAAAACTGGGCTACTACGTATCAAAAAGTGCCTGCTTTTATCGATGAAACGCTTGCCGCTAAAGGGGCAGAAAACATCGCTGACCGCGGTGAAGCAGATGCAAGCGACGACTTTGAAGGCACATATGAAGAATGGCGTGAACATATGTGGAGTGACGTAGCAGCCTACTTTAACCTCGACATTGAAAACAGTGAAGATAATAAATCTACTCTTTCACTTCAATTTGTCGACAGCGCCGCGGATATGCCGCTTGCGAAAATGCACGGTGCGTTTTCAACGAACGTCGTAGCAAGCAAAGAACTTCAACAGCCAGGCAGTGCACGAAGCACGCGACATCTTGAAATTGAACTTCCAAAAGAAGCTTCTTATCAAGAAGGAGATCATTTAGGTGTTATTCCTCGCAACTATGAAGGAATAGTAAACCGTGTAACAGCAAGGTTCGGCCTAGATGCATCACAGCAAATCCGTCTGGAAGCAGAAGAAGAAAAATTAGCTCATTTGCCACTCGCTAAAACAGTATCCGTAGAAGAGCTTCTGCAATACGTGGAGCTTCAAGATCCTGTTACGCGCACGCAGCTTCGCGCAATGGCTGCTAAAACGGTCTGCCCGCCGCATAAAGTAGAGCTTGAAGCCTTGCTTGAAAAGCAAGCCTACAAAGAACAAGTGCTGGCAAAACGTTTAACAATGCTTGAACTGCTTGAAAAATACCCGGCGTGTGAAATGAAATTCAGCGAATTTATCGCCCTTCTGCCAAGCATACGCCCGCGCTATTACTCGATTTCTTCATCACCTCGTGTCGATGAAAAACAAGCAAGCATCACGGTCAGCGTTGTCTCAGGAGAAGCGTGGAGCGGATATGGAGAATATAAAGGAATTGCGTCGAACTATCTTGCCGAGCTGCAAGAAGGAGATACGATTACGTGCTTTATTTCCACACCGCAGTCAGAATTTACGCTGCCAAAAGACCCTGAAACGCCGCTTATCATGGTCGGACCGGGAACAGGCGTCGCGCCGTTTAGAGGCTTTGTGCAGGCGCGCAAACAGCTAAAAGAACAAGGACAGTCACTTGGAGAAGCACATTTATACTTCGGCTGCCGTTCACCTCATGAAGACTATCTGTATCAAGAAGAGCTTGAAAACGCCCAAAGCGAAGGCATCATTACGCTTCATACCGCTTTTTCTCGCATGCCAAATCAGCCGAAAACATACGTTCAGCACGTAATGGAACAAGACGGCAAGAAATTGATTGAACTTCTTGATCAAGGAGCGCACTTCTATATTTGCGGAGACGGAAGCCAAATGGCACCTGCCGTTGAAGCAACGCTTATGAAAAGCTATGCTGACGTTCACCAAGTGAGTGAAGCAGACGCTCGCTTATGGCTGCAGCAGCTAGAAGAAAAAGGCCGATACGCAAAAGACGTGTGGGCTGGGTAA

**GDH:**

ATGTATACAGATTTAAAAGATAAAGTAGTAGTAATTACAGGTGGATCAACAGGTTTAGGACGCGCAATGGCTGTTCGTTTCGGTCAAGAAGAAGCAAAAGTTGTTATTAACTATTACAACAATGAAGAAGAAGCTTTAGATGCGAAAAAAGAAGTAGAAGAAGCAGGCGGACAAGCAATCATCGTTCAAGGCGACGTAACAAAAGAAGAAGATGTTGTAAACCTTGTTCAAACAGCTATTAAAGAATTCGGTACATTAGACGTTATGATTAATAACGCTGGTGTTGAAAACCCAGTTCCTTCTCATGAGTTATCTTTAGACAACTGGAATAAAGTTATTGATACAAACTTAACAGGTGCATTCTTAGGAAGCCGTGAAGCAATCAAATATTTTGTTGAAAACGACATTAAAGGAAACGTTATTAACATGTCTAGTGTTCATGAAATGATTCCTTGGCCATTATTTGTTCATTACGCAGCAAGTAAAGGCGGTATGAAACTAATGACGGAAACATTGGCTCTTGAATATGCGCCAAAAGGTATCCGCGTAAATAACATTGGACCAGGTGCGATGAACACACCAATTAACGCAGAGAAATTTGCAGATCCTGTACAACGTGCAGACGTAGAAAGCATGATTCCAATGGGTTACATCGGTAAACCAGAAGAAGTAGCAGCAGTTGCAGCATTCTTAGCATCATCACAAGCAAGCTATGTAACAGGTATTACATTATTTGCTGATGGTGGTATGACGAAATACCCATCATTCCAAGCAGGACGCGGATAA

**RBS:**

GGTACCTAAGGAGATATATC

**pRSFDuet-1:**

GGGGAATTGTGAGCGGATAACAATTCCCCTGTAGAAATAATTTTGTTTAACTTTAATAAGGAGATATACCATGGGCAGCAGCCATCACCATCATCACCACAGCCAGGATCCGAATTCGAGCTCGGCGCGCCTGCAGGTCGACAAGCTTGCGGCCGCATAATGCTTAAGTCGAACAGAAAGTAATCGTATTGTACACGGCCGCATAATCGAAATTAATACGACTCACTATAGGGGAATTGTGAGCGGATAACAATTCCCCATCTTAGTATATTAGTTAAGTATAAGAAGGAGATATACATATGGCAGATCTCAATTGGATATCGGCCGGCCACGCGATCGCTGACGTCGGTACCCTCGAGTCTGGTAAAGAAACCGCTGCTGCGAAATTTGAACGCCAGCACATGGACTCGTCTACTAGCGCAGCTTAATTAACCTAGGCTGCTGCCACCGCTGAGCAATAACTAGCATAACCCCTTGGGGCCTCTAAACGGGTCTTGAGGGGTTTTTTGCTGAAACCTCAGGCATTTGAGAAGCACACGGTCACACTGCTTCCGGTAGTCAATAAACCGGTAAACCAGCAATAGACATAAGCGGCTATTTAACGACCCTGCCCTGAACCGACGACAAGCTGACGACCGGGTCTCCGCAAGTGGCACTTTTCGGGGAAATGTGCGCGGAACCCCTATTTGTTTATTTTTCTAAATACATTCAAATATGTATCCGCTCATGAATTAATTCTTAGAAAAACTCATCGAGCATCAAATGAAACTGCAATTTATTCATATCAGGATTATCAATACCATATTTTTGAAAAAGCCGTTTCTGTAATGAAGGAGAAAACTCACCGAGGCAGTTCCATAGGATGGCAAGATCCTGGTATCGGTCTGCGATTCCGACTCGTCCAACATCAATACAACCTATTAATTTCCCCTCGTCAAAAATAAGGTTATCAAGTGAGAAATCACCATGAGTGACGACTGAATCCGGTGAGAATGGCAAAAGTTTATGCATTTCTTTCCAGACTTGTTCAACAGGCCAGCCATTACGCTCGTCATCAAAATCACTCGCATCAACCAAACCGTTATTCATTCGTGATTGCGCCTGAGCGAGACGAAATACGCGGTCGCTGTTAAAAGGACAATTACAAACAGGAATCGAATGCAACCGGCGCAGGAACACTGCCAGCGCATCAACAATATTTTCACCTGAATCAGGATATTCTTCTAATACCTGGAATGCTGTTTTCCCGGGGATCGCAGTGGTGAGTAACCATGCATCATCAGGAGTACGGATAAAATGCTTGATGGTCGGAAGAGGCATAAATTCCGTCAGCCAGTTTAGTCTGACCATCTCATCTGTAACATCATTGGCAACGCTACCTTTGCCATGTTTCAGAAACAACTCTGGCGCATCGGGCTTCCCATACAATCGATAGATTGTCGCACCTGATTGCCCGACATTATCGCGAGCCCATTTATACCCATATAAATCAGCATCCATGTTGGAATTTAATCGCGGCCTAGAGCAAGACGTTTCCCGTTGAATATGGCTCATACTCTTCCTTTTTCAATATTATTGAAGCATTTATCAGGGTTATTGTCTCATGAGCGGATACATATTTGAATGTATTTAGAAAAATAAACAAATAGGCATGCAGCGCTCTTCCGCTTCCTCGCTCACTGACTCGCTACGCTCGGTCGTTCGACTGCGGCGAGCGGTGTCAGCTCACTCAAAAGCGGTAATACGGTTATCCACAGAATCAGGGGATAAAGCCGGAAAGAACATGTGAGCAAAAAGCAAAGCACCGGAAGAAGCCAACGCCGCAGGCGTTTTTCCATAGGCTCCGCCCCCCTGACGAGCATCACAAAAATCGACGCTCAAGCCAGAGGTGGCGAAACCCGACAGGACTATAAAGATACCAGGCGTTTCCCCCTGGAAGCTCCCTCGTGCGCTCTCCTGTTCCGACCCTGCCGCTTACCGGATACCTGTCCGCCTTTCTCCCTTCGGGAAGCGTGGCGCTTTCTCATAGCTCACGCTGTTGGTATCTCAGTTCGGTGTAGGTCGTTCGCTCCAAGCTGGGCTGTGTGCACGAACCCCCCGTTCAGCCCGACCGCTGCGCCTTATCCGGTAACTATCGTCTTGAGTCCAACCCGGTAAGACACGACTTATCGCCACTGGCAGCAGCCATTGGTAACTGATTTAGAGGACTTTGTCTTGAAGTTATGCACCTGTTAAGGCTAAACTGAAAGAACAGATTTTGGTGAGTGCGGTCCTCCAACCCACTTACCTTGGTTCAAAGAGTTGGTAGCTCAGCGAACCTTGAGAAAACCACCGTTGGTAGCGGTGGTTTTTCTTTATTTATGAGATGATGAATCAATCGGTCTATCAAGTCAACGAACAGCTATTCCGTTACTCTAGATTTCAGTGCAATTTATCTCTTCAAATGTAGCACCTGAAGTCAGCCCCATACGATATAAGTTGTAATTCTCATGTTAGTCATGCCCCGCGCCCACCGGAAGGAGCTGACTGGGTTGAAGGCTCTCAAGGGCATCGGTCGAGATCCCGGTGCCTAATGAGTGAGCTAACTTACATTAATTGCGTTGCGCTCACTGCCCGCTTTCCAGTCGGGAAACCTGTCGTGCCAGCTGCATTAATGAATCGGCCAACGCGCGGGGAGAGGCGGTTTGCGTATTGGGCGCCAGGGTGGTTTTTCTTTTCACCAGTGAGACGGGCAACAGCTGATTGCCCTTCACCGCCTGGCCCTGAGAGAGTTGCAGCAAGCGGTCCACGCTGGTTTGCCCCAGCAGGCGAAAATCCTGTTTGATGGTGGTTAACGGCGGGATATAACATGAGCTGTCTTCGGTATCGTCGTATCCCACTACCGAGATGTCCGCACCAACGCGCAGCCCGGACTCGGTAATGGCGCGCATTGCGCCCAGCGCCATCTGATCGTTGGCAACCAGCATCGCAGTGGGAACGATGCCCTCATTCAGCATTTGCATGGTTTGTTGAAAACCGGACATGGCACTCCAGTCGCCTTCCCGTTCCGCTATCGGCTGAATTTGATTGCGAGTGAGATATTTATGCCAGCCAGCCAGACGCAGACGCGCCGAGACAGAACTTAATGGGCCCGCTAACAGCGCGATTTGCTGGTGACCCAATGCGACCAGATGCTCCACGCCCAGTCGCGTACCGTCTTCATGGGAGAAAATAATACTGTTGATGGGTGTCTGGTCAGAGACATCAAGAAATAACGCCGGAACATTAGTGCAGGCAGCTTCCACAGCAATGGCATCCTGGTCATCCAGCGGATAGTTAATGATCAGCCCACTGACGCGTTGCGCGAGAAGATTGTGCACCGCCGCTTTACAGGCTTCGACGCCGCTTCGTTCTACCATCGACACCACCACGCTGGCACCCAGTTGATCGGCGCGAGATTTAATCGCCGCGACAATTTGCGACGGCGCGTGCAGGGCCAGACTGGAGGTGGCAACGCCAATCAGCAACGACTGTTTGCCCGCCAGTTGTTGTGCCACGCGGTTGGGAATGTAATTCAGCTCCGCCATCGCCGCTTCCACTTTTTCCCGCGTTTTCGCAGAAACGTGGCTGGCCTGGTTCACCACGCGGGAAACGGTCTGATAAGAGACACCGGCATACTCTGCGACATCGTATAACGTTACTGGTTTCACATTCACCACCCTGAATTGACTCTCTTCCGGGCGCTATCATGCCATACCGCGAAAGGTTTTGCGCCATTCGATGGTGTCCGGGATCTCGACGCTCTCCCTTATGCGACTCCTGCATTAGGAAATTAATACGACTCACTATA

# Amino acid sequence information

**CYP109B1/** *Bacillus subtilis* 168

MNVLNRRQALQRALLNGKNKQDAYHPFPWYESMRKDAPVSFDEENQVWSVFLYDDVKKVVGDKELFSSCMPQQTSSIGNSIINMDPPKHTKIRSVVNKAFTPRVMKQWEPRIQEITDELIQKFQGRSEFDLVHDFSYPLPVIVISELLGVPSAHMEQFKAWSDLLVSTPKDKSEEAEKAFLEERDKCEEELAAFFAGIIEEKRNKPEQDIISILVEAEETGEKLSGEELIPFCTLLLVAGNETTTNLISNAMYSILETPGVYEELRSHPELMPQAVEEALRFRAPAPVLRRIAKRDTEIGGHLIKEGDMVLAFVASANRDEAKFDRPHMFDIRRHPNPHIAFGHGIHFCLGAPLARLEANIALTSLISAFPHMECVSITPIENSVIYGLKSFRVKM
